# A Capsid Protein Fragment of a Fusagra-like Virus Found in *Carica papaya* Latex Interacts with the 50S Ribosomal Protein L17

**DOI:** 10.3390/v15020541

**Published:** 2023-02-15

**Authors:** Marlonni Maurastoni, Tathiana F. Sá Antunes, Emanuel F. M. Abreu, Simone G. Ribeiro, Angela Mehta, Marcio M. Sanches, Wagner Fontes, Elliot W. Kitajima, Fabiano T. Cruz, Alexandre M. C. Santos, Jose A. Ventura, Ana C. M. M. Gomes, F. Murilo Zerbini, Patricia Sosa-Acosta, Fábio C. S. Nogueira, Silas P. Rodrigues, Francisco J. L. Aragão, Anna E. Whitfield, Patricia M. B. Fernandes

**Affiliations:** 1 Biotechnology Core, Federal University of Espírito Santo, Vitória 29043-900, ES, Brazil; 2 Department of Entomology and Plant Pathology, North Carolina State University, 840 Main Campus Drive, Raleigh, NC 27606, USA; 3 Embrapa Recursos Genéticos e Biotecnologia, Brasília 70770-917, DF, Brazil; 4 Department of Cell Biology, University of Brasilia, Brasilia 70910-900, DF, Brazil; 5 Department of Phytopathology, University of São Paulo, Piracicaba 13418-900, SP, Brazil; 6 Espírito Santo Institute for Research, Technical Assistance and Rural Extension, Vitória 29052-010, ES, Brazil; 7 Department of Phytopathology, Federal University of Viçosa, Viçosa 36570-900, MG, Brazil; 8 Department of Biochemistry, Institute of Chemistry, Federal University of Rio de Janeiro, Rio de Janeiro 21941-909, RJ, Brazil; 9 Multidisciplinary Core for Research in Biology, Campus Duque de Caxias, Federal University of Rio de Janeiro, Duque de Caxias 25240-005, RJ, Brazil

**Keywords:** *Totiviridae*, fusagra-like virus, protein–protein interaction, coat protein, dsRNA, plant virus

## Abstract

Papaya sticky disease is caused by the association of a fusagra-like and an umbra-like virus, named papaya meleira virus (PMeV) and papaya meleira virus 2 (PMeV2), respectively. Both viral genomes are encapsidated in particles formed by the PMeV ORF1 product, which has the potential to encode a protein with 1563 amino acids (aa). However, the structural components of the viral capsid are unknown. To characterize the structural proteins of PMeV and PMeV2, virions were purified from *Carica papaya* latex. SDS-PAGE analysis of purified virus revealed two major proteins of ~40 kDa and ~55 kDa. Amino-terminal sequencing of the ~55 kDa protein and LC-MS/MS of purified virions indicated that this protein starts at aa 263 of the deduced ORF1 product as a result of either degradation or proteolytic processing. A yeast two-hybrid assay was used to identify *Arabidopsis* proteins interacting with two PMeV ORF1 product fragments (aa 321–670 and 961–1200). The 50S ribosomal protein L17 (*At*RPL17) was identified as potentially associated with modulated translation-related proteins. In plant cells, *At*RPL17 co-localized and interacted with the PMeV ORF1 fragments. These findings support the hypothesis that the interaction between PMeV/PMeV2 structural proteins and RPL17 is important for virus–host interactions.

## 1. Introduction

Papaya sticky disease (PSD) is an economically important problem in papaya production in Brazil, Mexico, and Australia, and has been referred to as “meleira” in Brazil. Its causal agent was initially identified as a double-stranded (ds) RNA virus with an isometric particle of approximately 50 nm in diameter, named papaya meleira virus (PMeV) [1,2]. A second positive-sense single-stranded ((+) ssRNA) virus, designated as papaya meleira virus 2 (PMeV2), was also proved to be an important agent in the onset of symptoms associated with PSD [3] (reviewed in [4]). These two viruses make up the PMeV complex, an association of a virus tentatively classified in the proposed *Fusagraviridae* family [5,6] (PMeV) and an umbra-like virus (PMeV2) [3,7]. The PMeV complex accumulates in *Carica papaya* laticifers [1], a living plant structure specialized in the storage of latex, a complex fluid enriched in proteases [8,9]. Within these cells, the PMeV complex promotes spontaneous latex exudation, which results in a sticky appearance of papaya fruit after oxidation. So far, no vector has been convincingly identified.

Similar to the virus–virus interaction between the dsRNA yadokari virus 1 (YkV1) and the (+) ssRNA yadonushi virus 1 (YnV1) [6,10], a trans-encapsidation phenomenon is observed for the PMeV complex as both viruses are encapsidated by the PMeV genome-encoded capsid protein (CP)—the translation product of PMeV open reading frame (ORF) 1 [3]. The full-length PMeV ORF1 is predicted to encode a protein of 1563 amino acids (aa) which is 20–26% identical to analogous proteins found in mycoviruses but has no significant matches with other proteins. Only 8% of the PMeV ORF1 predicted amino acid sequence has been experimentally validated after virion purification from *C. papaya* latex by sucrose gradient followed by mass spectrometry analysis of the isolated proteins. Nine peptides were identified matching PMeV ORF1 predicted amino acid sequence (125 of 1563 amino acids) encompassing the central region of the putative protein (from aa 356 to 785) [3].

Additional functions have been reported for CPs of plant viruses other than the protection of the viral genome, such as participating cell-to-cell and systemic movement, being a component of genome transcription and replication complexes, modulating host defense pathways, and processing host mRNA [11,12,13,14]. CP homodimers are the building blocks of a specific type of icosahedral capsid, mainly found among dsRNA viruses, usually needed to avoid host cell defense mechanisms. This T = 1 capsid, comprised of 120 subunits of 60 asymmetrical dimers (also known as a T = 2 layer capsid), is found in members of the families *Reoviridae*, *Picobirnaviridae*, and *Cystoviridae*, and in the mycoviruses of the families *Totiviridae*, *Partitiviridae*, and *Megabirnaviridae* [15]. Mycovirus capsid proteins share a conserved α-helical domain, even though they are not similar in sequence, and insertions near this domain seem to allow the acquisition of enzymatic functions [15,16].

PMeV is phylogenetically related to a number of fungi- and insect-infecting, non-segmented, dsRNA viruses with two ORFs [3]. These viruses share similar features with members of the *Totiviridae* family, e.g., a 3′ proximal ORF predicted to express an RNA-dependent RNA polymerase (RdRp) through a -1 ribosomal frameshifting and a 5′ ORF encoding structural proteins. However, they are only distantly phylogenetically related to totivirids, and have been proposed to comprise a new family named *Fusagraviridae* which would most likely be classified in the order *Ghabrivirales* (which includes *Totiviridae*) [5]. Thus, we will henceforth refer to PMeV as a “fusagra-like” virus.

Our main understanding of how plants respond to fusagra-like viruses comes from the PSD pathosystem, which has been studied at the biochemical and molecular levels [17,18,19,20]. Proteomic and transcriptomic analysis of infected *C. papaya* shows a symptoms tolerance mechanism before flowering, mainly related to changes in hormone-responsive genes, protein turnover, and chloroplast-related proteins. The PMeV complex also perturbs proteins in the laticifers, where levels of cysteine proteases and serine protease inhibitors are reduced. Although we know the infection effects on the accumulation of proteins and transcripts, information about key aspects of the plant–virus interaction and data on the viral protein–plant protein interactions, essential for developing more effective strategies to control PSD, are still missing.

We report herein a detailed analysis of the structural protein coding region of PMeV. PMeV virion components separate into two major proteins, p40 and p55 (named after their relative molecular masses), with p55 being the PMeV ORF1 translation product processed or degraded at amino acid position 263. We sought to identify Arabidopsis proteins that interact with two fragments of PMeV ORF1 by using a yeast two-hybrid (Y2H) assay, which identified 28 interacting proteins mostly targeted to the chloroplast. We also built a protein–protein interaction (PPI) network showing that PMeV capsid protein(s) can be indirectly responsible for modulating the expression of several proteins during the pre-and post-flowering development stages of infected *C. papaya*. The Y2H assay and the PPI network support the idea that the 50S ribosomal protein L17 (RPL17), a CP-interacting protein, can be an important target to modulate virus infection.

## 2. Materials and Methods

### 2.1. Virus Purification and Transmission Electron Microscopy Analysis

Virions of the PMeV complex were purified from *C. papaya* latex using a sucrose gradient [3]. Latex was collected from papaya fruits in 0.1 mol·L^−1^ sodium citrate pH 5.0 supplemented with 10 µmol·L^−1^ E-64 (L-trans-3-Carboxyoxiran-2-carbonyl-l-leucylagmatine) (1 latex: 1 sodium citrate, *v/v*). Then, 2 volumes of 0.1 mol·L^−1^ ammonium citrate solution pH 6.5 supplemented with 0.037 mol·L^−1^ iodoacetamide, 0.15 mol·L^−1^ NaDIECA (Sodium diethyldithiocarbamate trihydrate) and 100 µg·ml^−1^ PMSF (phenylmethylsulfonyl fluoride) were added to the latex: sodium citrate solution. The mixture was centrifuged at 3800× *g* for 10 min at 4 °C and the supernatant was clarified for 3 h with Triton X-100 3% (*v/v*). The clarified mixture was transferred to 30 mL polycarbonate tubes and ultracentrifuged at 100,000× *g* for 90 min at 4 °C in a fixed angle rotor, through a 20% (*w/v*) sucrose cushion (4 clarified mixture: 1 sucrose cushion, *v/v*). Pellets were resuspended in 500 µL of 0.01 mol·L^−1^ borate buffer, pH 9.0, and the purified virus was stored overnight at 4 °C. In parallel, 1.4 mL of 10–40% (*w/v*) sucrose solutions, prepared in 0.01 mol·L^−1^ borate buffer pH 9.0, were mounted in an 8 mL polycarbonate tube and allowed to form a linear gradient overnight at 4 °C. Viral preparations were overlayered on the sucrose gradient and centrifuged at 114,000× *g* for 90 min at 4 °C in a swing bucket rotor. The three light-scattering fractions [3] were collected, combined, and layered onto a 50% (*w/v*) cesium chloride solution (prepared in 0.01 mol·L^−1^ borate buffer pH 9.0) followed by ultracentrifugation at 145,000× *g*, for 18 h at 4 °C in a swing bucket rotor (adapted from [21]). The low- and high-density fractions containing virions (M and B, respectively) were collected and centrifuged again at 35,000× *g*, for 3.5 h in a fixed-angle rotor. The final pellets were resuspended in 0.01 mol·L^−1^ borate buffer, pH 9.0. The pellets were negatively stained in 2% (*w/v*) potassium phosphotungstate, pH 6.8, and observed in a JEOL JEM-1011 transmission electron microscope (TEM).

### 2.2. Protein Identification of Virion Preparations

#### 2.2.1. Tandem Mass Spectrometry

The cesium chloride-purified virions (3–7 μg of protein) were resuspended in a 4:1 (*w/w*) urea/thiourea solution. The samples were incubated with 10 mmol·L^−1^ DTT (Sigma-Aldrich, St. Louis, MO, USA) at 37 °C for 1 h and then with 40 mmol·L^−1^ iodoacetamide (Bio-Rad, Hercules, CA, USA) for 30 min at room temperature in the dark. Samples were diluted 5 times with 100 mmol·L^−1^ Tris (hydroxymethyl) aminomethane (Bio-Rad, Hercules, CA, USA) buffer, pH 8.3. For protein digestion, trypsin (Promega, Madison, WI, USA) was mixed in a 1:25 ratio (enzyme: substrate) and incubated at 25 °C for 24 h. Tryptic peptides were desalted in Macro SpinColumns C18 (Harvard Apparatus, Holliston, MA USA), dried in a vacuum concentrator (Martin Christ, Germany), and resuspended in 0.1% formic acid. The peptide concentration was determined through Qubit Assay (Invitrogen, Waltham, MA, USA) following the manufacturer’s instructions.

Tryptic peptides were analyzed in two technical replicates in a Q-Exactive Plus mass spectrometer (Thermo Fisher Scientific, Waltham, MA, USA) coupled to an Easy-1000 nLC system (Thermo Fisher Scientific, Waltham, MA, USA). The trap-column was an Acclaim™ PepMap™ 100 C18 HPLC Columns with 20 mm (3 μm spheres and 75 μm ID, Thermo Scientific, Waltham, MA, USA) and the analytical column was an Easy-Spray Column (2 μm spheres and 75 μm ID, reverse-phase (Thermo Fisher Scientific, Waltham, MA, USA) with 25 cm. The mobile phases were 5% ACN/0.1% formic acid (Solvent A) and 95% ACN/0.1% formic acid (Solvent B). The peptide mixture was separated with the following gradient: 5–25% B for 20 min, 25–50% B for 15 min, 50–60% B for 10 min, 60–80% B for 10 min, 80–95% B for 15 min, and 95% B for 20 min at 300 nL·min^−1^ of flow rate at 50 °C.

Tandem mass spectrometry was performed with the data-dependent acquisition method and positive polarity. For the analysis, the following full scan parameters were used: 1 Microscan, 70,000 resolutions at *m/z* 200, AGC target of 3E6 ions, 50 ms maximum IT, and the range of mass acquired was 350–2000 *m/z*. MS2 parameters were 17,500 resolutions at *m/z* 200, AGC target of 2E5 ion, 100 ms maximum IT, loop count of 20, 2.0 Th of isolation window, minimum intensity threshold of 100,000 ions equipped with high-energy collision dissociation (HCD) using a normalized collision energy of 30%, a dynamic exclusion list of 45 s, and spray voltage at 1.9 Kv.

Proteome Discoverer software version 2.1 (Thermo Fisher Scientific, Waltham, MA, USA) was employed for the raw data processing. Protein identification was carried out according to Quiñones-Vega et al. [22]. Three protein databases were used (all downloaded in September 2022): (i) *Arabidopsis thaliana* UniProt database (https://www.uniprot.org/uniprotkb?query=(taxonomy_id:3699)%20AND%20(taxonomy_id:3702)%20OR%20(taxonomy_id:3649), accessed on 29 September 2022), (ii) plant viruses + taxonomy_id:33090 + PMeV from Uniprot database (https://www.uniprot.org/uniprotkb?query=virus%20AND%20(taxonomy_id:33090), accessed on 29 September 2022), and (iii) *Carica papaya* NCBI database (*Carica papaya*) AND “*Carica papaya*”[porgn:__txid3649]. In addition, a new sequence of the PMeV ORF1 which was cloned from infected latex (this work) was also added to all three databases. Peptide-spectrum match (PSM) validation was assessed through a fixed value, considering PSM with a Maximum Delta Cn of 5%. The false discovery rate was <1% at PSM, peptide, and protein levels. For label-free quantification, the area of up to the three most abundant unique and razor peptides was used. Proteins were grouped into protein groups using the maximum parsimony principle.

#### 2.2.2. Amino-Terminal Sequencing

The protein samples were also submitted to N-terminal automatic amino acid sequencing. After sodium dodecyl sulfate–polyacrylamide gel electrophoresis (SDS-PAGE), the separated proteins were transferred from the gel to a Sequi-blot polyvinylidene fluoride (PVDF) membrane for protein sequencing, followed by Coomassie brilliant blue R-250 staining according to the manufacturer’s instruction (Bio-Rad, Hercules, CA, USA). Then, the protein bands at 50 kDa molecular mass were excised from the membrane, processed, and sequenced on a Shimadzu PPSQ-33A. Chromatograms were analyzed using the PPSQ-30 Data Processing version 1.10 (Shimadzu, Kyoto, Japan).

### 2.3. P55 Structural Homology, Domain and CLEAVAGE Prediction

A structural model for p55 (aa 263–739 GenBank accession OP834191) was built using AlphaFold [23] accessed in the Google collaborative AlphaFold notebook (https://colab.research.google.com/github/sokrypton/ColabFold/blob/main/AlphaFold2.ipynb, accessed on 25 December 2022). All parameters were set to the default except the number of generated models and the maximum number of recycles, which were kept at 3 and 48, respectively. The best rank model was used for a structural alignment with all proteins in the database that have structural-resolved proteins using the DALI server [24]. Structure superimposition was carried out using UCFS Chimera [25]. InterProScan was used to identify and classify protein families [26]. The 2A-like or pseudo 2A-sites were manually searched in the ORF1-encoded protein [27]. Peptide Cutter software from ExPASy (https://www.expasy.org/, accessed on 5 January 2023) was used for the prediction of protease cleavage sites in the ORF1-encoded protein.

### 2.4. Cloning of PMeV ORF1 and AtRPL17

To generate a PMeV ORF1 clone, total RNA was extracted from 100 µL of a pool of latex from sticky diseased *C. papaya* using TRIzol reagent (Thermo Fisher Scientific, Waltham, MA, USA), according to the manufacturer’s protocol. One microgram of total RNA treated with DNAse I (Thermo Fisher Scientific, Waltham, MA, USA) was used for cDNA synthesis using Superscript III Reverse Transcriptase and the sequence-specific reverse primer according to the manufacturer’s instructions. PCR was performed using Platinum High Fidelity DNA Polymerase (Invitrogen, Waltham, MA, USA) and specific primers following the manufacturer’s instructions in a Mastercycler Thermocycler (Eppendorf, Hamburg, Germany). PCR amplicon was visualized on 1% (*w/v*) agarose gel, excised, and purified using PureLink Quick Gel Extraction Kit (Thermo Fisher Scientific, Waltham, MA, USA). The amplicon was cloned into Gateway pDONR™221 vector, and the whole clone was submitted to Sanger sequencing. The clone was named pDONR221-PMeVORF1-ES (GenBank accession OP834191). To generate PMeV ORF1 fragments, a total of 10 primer pairs were designed based on the pDONR221-PMeVORF1-ES sequence. In addition, AtRPL17 full-length gene was amplified from a pGADT7 plasmid recovered from an Arabidopsis cDNA library in yeast. The plasmid was sequenced and compared with the NCBI database. Amplicons from both PMeV ORF1 fragments and AtRPL17 were cloned into pENTR-D/TOPO and then recombined to yeast and plant expression vectors using Gateway cloning techniques. Primers used in this work are listed in Table S1.

### 2.5. Binary Interactions and Library Screening Using Yeast Two-Hybrid Assay

A yeast two-hybrid system (Y2H) was used to test binary interactions between PMeV ORF1 fragments. We recombined a pENTR-D/TOPO clone containing a PMeV ORF1 fragment with the pDESTGADT7 and pDESTGBKT7 destination vectors to generate a protein fused to the GAL4 activation domain (AD) and binding domain (BD). Briefly, the yeast reporter strain Y2HGold (Takara Bio Inc., Kusatsu, Shiga, Japan) was co-transformed with both AD and BD plasmids according to the manufacturer’s instructions. Co-transformants were selected by culture on double dropout media (DDO), i.e., synthetic defined minimal media (SD) lacking leucine and tryptophan (SD/-L/-W). Positive interactions were selected by culture on quadruple dropout media (QDO), i.e., SD lacking leucine, tryptophan, histidine, and adenine (SD/-L/-W/-H/-A). We co-transformed plasmids containing GAL4 DNA-BD fused with murine p53 (pGBKT7-p53) plus GAL4 AD fused with SV40 large T-antigen (pGADT7-T) as a positive control and GAL4 BD fused with lamin (pGBKT7-Lam) plus pGADT7-T as a negative control. We also tested the autoactivation of each fragment by co-transforming BD and AD plasmids with empty plasmids. An overnight culture of all co-transformants was normalized to OD_600_ 2, spotted in DDO and QDO/X/A (QDO media supplemented with Aureobasidin A and the chromogenic substrate X-alpha-gal) plates, and kept at 30 °C for 4 days. The entire experiment was performed three times.

Y2H library screening was performed using the Matchmaker Mate & Plate Two-Hybrid System (Takara Bio Inc., Kusatsu, Shiga, Japan). We used this system because the library is normalized, reducing the amount of redundant proteins for the screen and due to the better characterization of Arabidopsis proteins when compared to papaya proteins. To choose the bait, we performed an expression assay in yeast following the manufacturer’s instructions. Plasmids containing the fragments fused to GAL4 BD were transformed in the yeast strain Y2HGold according to the manufacturer’s protocol and spread on single dropout media (SDO), i.e., SD lacking tryptophan (SD/-W). Protein expression was induced by growing the positive transformants on YPD media. Yeast proteins were extracted according to Kushnirov [28] and normalized to the equivalent of 2–3 OD_600_ units. The expression of fused proteins was verified by SDS-PAGE and Western blotting. Briefly, proteins were transferred to a PVDF membrane using the Trans-Blot Turbo RTA Transfer Kit in a Trans-Blot Turbo transfer system (Bio-Rad, Hercules, CA, USA). Next, membranes were incubated overnight in blocking buffer (5% nonfat milk in Tris-buffered saline with 0.5% Tween [TBS-T]) followed by incubation with mouse anti-myc monoclonal antibody (Invitrogen, Waltham, MA, USA) at 1:5000 dilution for 1.5 h. Then, membranes were washed two times for 15 min with TBS-T followed by incubation with a goat anti-mouse IgG (H + L)-HRP Conjugated (Bio-Rad, Hercules, CA, USA) at 1:10,000 dilution. Finally, membranes were washed three times for 10 min with TBS-T and developed with 200 μL of Prometheus ProSignal Pico chemiluminescent substrate for 5 min. The membrane was photographed using the iBright CL1000 imager (Thermo Fisher Scientific, Waltham, MA USA).

A normalized cDNA Arabidopsis library fused to GAL4 AD in the yeast strain Y187 (Mate & Plate Library–Takara Bio Inc., Kusatsu, Shiga, Japan) was used to mate with Y2HGold containing the ORF1 fragment 4 (CP4, aa 961–1200) fused to GAL4 BD. Cells were initially screened on 60 150 mm plates containing DDO/X/A media. Then, 306 blue colonies were patched on QDO/X/A media and QDO/X/A media supplement with 2.5 mmol·L^−1^ and 5 mmol·L^−1^ of 3-Amino-1,2,4-triazole (3-AT). Plasmids from colonies picked from all media were extracted from yeast, recovered in *Escherichia coli*, sequenced, and identified.

A total of 28 unique plasmids were transformed in yeast with an empty GAL4 BD plasmid and ORF1 fragment 4 AD-containing plasmid to test autoactivation and interaction, respectively. Given that most of our peptides obtained in the mass spectrometry analysis covered ORF1 fragment 2 (CP2, aa 321–670), we took advantage of the interaction between CP4 and CP2 (see results section) and also tested the 28 unique plasmid interactions with ORF1 fragment 2. Positive interactors were spotted in DDO, QDO/X/A, and QDO/X/A supplemented with 0 mmol·L^−1^, 1 mmol·L^−1^, 2.5 mmol·L^−1^, and 5 mmol·L^−1^ of 3–AT media. The interaction of pGBKT7-p53 and pGADT7-T was used as the positive control and pGBKT7-Lam and pGADT7-T as the negative control. The experiment was performed three times.

### 2.6. Protein–Protein Interaction Network

A protein–protein interaction network was constructed with differentially accumulated proteins of PMeV complex-infected *C. papaya* at 4- and 7-months post-germination [19], and CP2 and CP4-interacting proteins. Biomart (https://phytozome.jgi.doe.gov/biomart/, accessed on 22 April 2021) was used to obtain Arabidopsis orthologues from the Phytozome database (http://phytozome.jgi.doe.gov/, accessed on 22 April 2021). Sequences were uploaded on String [29] and an interaction network of high confidence level was exported to Cytoscape [30].

### 2.7. Transient Expression and Detection of Protein Interaction in Nicotiana Benthamiana

To confirm the interaction of PMeV capsid protein and RPL17 in vivo we used *N. benthiamana* as a plant cell system due to lack of an efficient system for heterologous protein expression in green tissues or laticifer cells of papaya. We first aimed to localize RPL17 and PMeV ORF1 fragment 2 in plant cells fusing proteins to red (RFP) or green fluorescent proteins (EGFP). For EGFP fusions, we used the binary plasmids pSITE-2CA and pSITE-2NB, and for RFP fusions, we used pSITE-4CA and pSITE-4NB. “-CA” or “-NB” indicates that the plasmid allows cloning of fluorescent protein fused to the N-terminus and C-terminus of the desired protein, respectively [31]. For the bimolecular fluorescence complementation (BiFC) assay, we recombined AtRPL17 and ORF1 fragment 2 into the BiFC vectors (pSITE-nen, pSITE-cen, pSITE-nec, pSITE-cec) as fusions either the C- or N-terminal sequence of both halves of the yellow fluorescent protein (eYFP) gene [32]. As a negative control, we challenged ORF1 fragment 2 with Glutathione S-transferase (GST) fusions to YFP halves. A positive interaction was considered if the fluorescence was above the signal observed for the negative control and if at least 50 cells with similar localization signals for the interaction were visualized. For *Agrobacterium*-mediated expression, an overnight culture of *A. tumefaciens* cells containing the plasmids was inoculated in a fresh media containing the appropriate antibiotics and brought to standard concentration (OD_600_ 0.5–1.0) in 10 mmol·L^−1^ MES buffer, pH 5.6, containing 10 mmol·L^−1^ MgCl_2_. The culture was incubated for at least 2 h at 28 °C with no agitation in the presence of 200 μmol·L^−1^ acetosyringone (Sigma-Aldrich, St. Louis, MO, USA).

Agroinfiltration was performed using 1 mL syringes without a needle on the abaxial side of *N. benthamiana* leaves wild type or transgenic expressing the histone 2B protein fused to RFP [32]. Leaves were analyzed for five days after agroinfiltration. Transient expression and localization of fluorescent fusion proteins, EGFP, RFP, or YFP in leaf cells of *N. benthamiana* were visualized using Cytation 5 image reader (BioTek, Winooski, VT, USA). Images were analyzed in Gen5 version 3.04 microplate reader and imager software (BioTek, Winooski, VT, USA). This experiment was performed three times, with at least two leaves per infiltration treatment.

## 3. Results

### 3.1. Identification of the PMeV Capsid-Protein Coding Region

**Figure 1:**
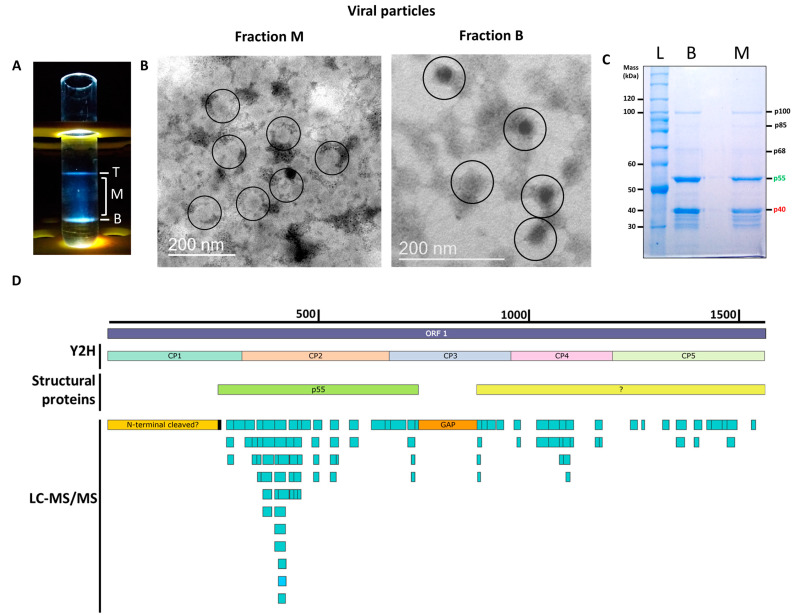
Characterization of the papaya meleira virus (PMeV) complex capsid protein composition. (**A**) The three opalescent fractions (T- Top, M- middle, and B- Bottom fraction) were obtained after ultracentrifugation at 145,000× *g* for 18 h at 4 °C in a 50% (*w/v*) cesium chloride isopycnic gradient. (**B**) Transmission electron microscopy images of viral particles from the M and B fractions. Viral particles are approximately 50 nm in diameter and are circled in the figure. (**C**) Coomassie blue-stained SDS-PAGE of fractions, lanes B and M are fractions from ultracentrifugation and L is the protein ladder. (**D**) Mapping positions of peptides identified in cesium chloride-purified virions collected from B and M fractions matching with the PMeV deduced ORF1 protein (GenBank accession OP834191) (light-blue rectangles) and protein fragments used for binary interactions by yeast two-hybrid (Y2H) and structural proteins. The black rectangle represents the N-terminal sequencing of the ~55 kDa band obtained after the separation of proteins in the virion preparation; the orange rectangle indicates an ~15 kDa gap with no peptides identified. The yellow rectangle represents a degraded or cleaved ~30 kDa N-terminal protein. The green rectangle represents p55. The light-yellow rectangle represents the putative coding region of uncharacterized structural protein(s).

To determine the structural proteins that form the PMeV capsid, virions were purified using sucrose gradient centrifugation [3]. As expected, three opalescent zones were visible. The additional cesium chloride isopycnic gradient purification yielded two well-defined bands at the tube top (T) and bottom (B), while a blurred zone appeared in the middle (M) of the tube (Figure 1A). The presence of PMeV complex virions was confirmed by the visualization of viral particles by transmission electron microscopy (Figure 1B). Fraction M contained a higher number of particles compared to fraction B, which showed a fibrillary material associated with them. No viral particle was obtained for the top fraction. Protein components of purified virions from both B and M fractions were separated by SDS-PAGE into two major proteins with estimated molecular masses of ~55 kDa and ~40 kDa, henceforth referred to as p55 and p40 (Figure 1C). Three minor bands of higher molecular mass, named as p68, p85, and p100, and several minor bands below p40 were also visualized. We were able to determine the N-terminal amino acid sequence of the p55 to be (S/G)XEQL(A)I (Figure S1), which mapped to positions 263 to 269 of the deduced ORF1 protein.

The LC-MS/MS analysis of tryptic peptides of purified virions from combined fractions M and B identified a total of 100 unique peptides for PMeV ORF1 and 5 peptides for PMeV ORF2 (45.8% and 6.2% coverage, respectively). No peptide fragments were assigned to PMeV2 ORF-encoded proteins. The first identified peptide matching with PMeV ORF1 (RNKAIPEYNTILVPQVK) starts at position 283, and the last identified peptide (EAHISQDQLR) ends at position 1541. The region with the highest coverage spans from position 283 to 731, where 61 peptides were identified (Figure 1D; Table S2).

### 3.2. PMeV p55 Share Conserved Domains with Mycovirus Capsid Proteins

We were able to use AlphaFold to generate a model for p55 (aa 263–739) with a predicted local distance difference test (pLDDT) of 94.7, an average of 66.3, and a minimum of 23.9, and a pTMscore of 0.7464 (Figure S2). The model did not have good reliability average pLDDT (70–90), either as result of p55 features or AlphaFold limitations. We used the model for a structural alignment with all proteins in the database that have been structurally resolved. The four most similar proteins are capsid proteins from (in decreasing order of similarity): Penicillium chrysogenum virus (PcV, family *Chrysoviridae*), Saccharomyces cerevisiae virus L-A (ScV L-A, family *Totiviridae*), Omono river virus (OmRV, tentatively classified in the *Totiviridae* family) and Trichomonas vaginalis virus 2 (TVV, family *Totiviridae*) (Table S3). We superimposed the PcV CP domain A with p55 and found four alpha helices that show close relative spatial positions (a11 with a7; a16 with a9; a21 with a10; a22 with a11) as well as small beta sheets (b5 with b5; b6 with b6; b7 with b9 and b8 with b10) of PcV CP domain A and p55, respectively (Figure S3). When the CPs of the three viruses, PcV, ScV L-A and OmRV are superimposed with the p55 model, a conserved structure composed of helix-turn-helix as well as two small alpha-helices show close relative spatial positions (Figure S4). We tried the same analysis using iTasser, but no homology with viral proteins was found.

InterProScan predictions revealed in the full length ORF1 product three low-complexity regions extending from aa 105 to 125, 214 to 227, and 1260 to 1270. No 2A-like sequences or pseudo 2A-sites were found in the PMeV ORF1 product, and no other domains were found in the protein databases accessed.

We predicted the cleavage sites that result in p55 using the ExPASy tool Peptide Cutter. In the p55 N-terminal region (aa 262 and 263), putative cleavage sites for Asp-N endopeptidase + N-terminal Glu, Pepsin pH 1.3, Pepsin pH > 2, proteinase K and Thermolysin were identified. In its C-terminal region (aa 739 and 740), putative cleavage sites for Lysyl endopeptidase and Trypsin were found.

### 3.3. A Fragment of p55 (CP2, aa 321–670) Interacts with a Viral Structural Protein Fragment, CP4 (aa 961–1200)

**Figure 2:**
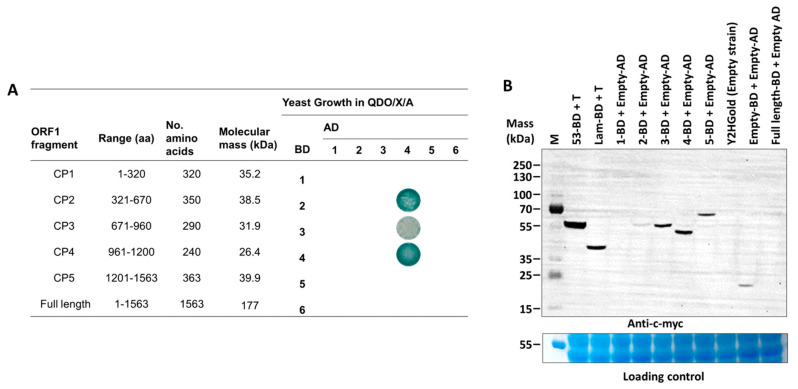
Summary of yeast two-hybrid assays mapping dimerization fragments in the papaya meleira virus (PMeV) ORF1 product, and detection of five fragments of the PMeV ORF1 product. (**A**) Each ORF1 fragment was fused either to the GAL4 binding domain (BD) or GAL4 activation domain (AD) in pDESTGBKT7 and pDESTGADT7, respectively, and transformed in the yeast strain Y2HGold. Positive interactors were selected in QDO/X/A media. (**B**) The c-myc-fused protein expression was verified by SDS-PAGE of yeast crude protein extracts and Western blotting using an anti-c-myc antibody. The 1- to 5-BD represents each fragment of the PMeV ORF1 product fused to GAL4 BD. 53-BD (GAL4 DNA-BD fused with murine p53) and Lam-BD (GAL4 BD fused with Lamin) were used as controls. Untransformed yeast and yeast transformed with pDEST-BGKT7 plasmids were used as the negative control. M: PageRuler Plus prestained protein ladder.

To determine dimerization regions on the PMeV capsid protein, we tested binary interactions of the full-length and five non-overlapping fragments of the PMeV ORF1 product using the yeast two-hybrid system (Figure 2A). ORF1 fragments for expression in yeast were selected based on secondary structure—we tried not to disrupt predicted alpha helices or beta strands (i.e., the fragments start and end at random coil regions). The five non-overlapping fragments tested were named CP1 (aa 1 to 320), CP2 (aa 321 to 670), CP3 (aa 671–960), CP4 (aa 961–1200), and CP5 (aa 1201–1563). Binary interactions were identified between CP2 and CP4, CP3 and CP4, and a self-interaction for CP4. No interactions were identified for the full-length protein in any combinations, or involving the CP1 and CP5 fragments.

### 3.4. Translation and RNA-Silencing Complexes Co-Purify with the PMeV Complex

To identify the occurrence of protein complexes associated with the PMeV complex virion, LC-MS/MS was used to analyze tryptic products of cesium chloride-purified virions from infected latex. Metrics of the identified proteins are shown in Table S4. Proteins with “Score Sequest HT” higher than the average and proteins with ≥2 unique peptides were considered to generate the final data set (Score Sequest HT is the sum of the scores of the individual peptides displayed by the Proteome Discoverer software; the higher this score, the higher the individual scores of the peptides, and thus the better the identification). Among redundant proteins, those with the highest “Score Sequest HT” were selected for the final dataset. Analysis using a *C. papaya* database was carried out similarly, but all values of “Score Sequest HT” were considered. When peptides were searched with a plant virus database, 32 unique plant proteins were identified, including proteins involved in translation regulation (A0A0P0INT0, A0A6G9KE37, A0A072VPN5, A0A0D3BWP8), mRNA processing (A0A5B8MBX6, A0A438F2I9), and RNA silencing (D3GBV0, A0A176VLC9, A0A343J650, A0A1R3HJK2, A0A072VPN5, W9QFR0, A0A6J1JYH8, A0A1U8PQK2, A0A103XMT4, A0A1S2XD67, A0A6J1BR97, D7M068, A0A2U1NM76, A0A5N5FGW1, A0A4D6NTN9, A0A1J3FIA8). When using a *C. papaya* database, five proteins were retrieved after removing redundant and uncharacterized proteins. These included three proteases (XP_021912319.1, CAA54974.1, P10056.2), one endochitinase (3CQL), and one pathogenesis-related protein 4 (4JP7) (Table S5).

### 3.5. Arabidopsis Library Screening Using a C-Terminal PMeV ORF1 Fragment (aa 961–1200) Identified 28 Plant-Interacting Proteins

A commercial Arabidopsis cDNA library was used to identify host proteins interacting with PMeV ORF1 product fragments. The best candidate to use as bait was determined after testing the expression of each fragment fused to the GAL4-binding domain (Figure 2B). We decided to use CP4 as bait and validate the interactors with both CP4 and CP2 fragments as CP4 showed a higher expression level compared to CP2 and given that CP4 interacts with CP2 in our binary yeast two-hybrid assay. Thus, using a GAL4-based yeast two-hybrid system, independent yeast transformants were screened using CP4 as bait. In total, 306, 36, and 21 colonies were screened from QDO/X/A supplement with 0 mmol·L^−1^, 2.5 mmol·L^−1^, and 5 mmol·L^−1^ of 3-AT, respectively. From 48 colonies sampled and sequenced from all three stringency conditions, 28 represented characterized and unique proteins interacting with CP2 and CP4 (Table 1; Figure S6). A Blast analysis shows that these proteins are 42–92% identical to their papaya orthologs (Table S6). Functional categorization showed that most proteins were targeted to the chloroplast and presented protein binding and catalytic activity.

### 3.6. CP2 Putatively Associates with Translation-Related Proteins Differentially Expressed in Pre-Flowering C. papaya

**Figure 3:**
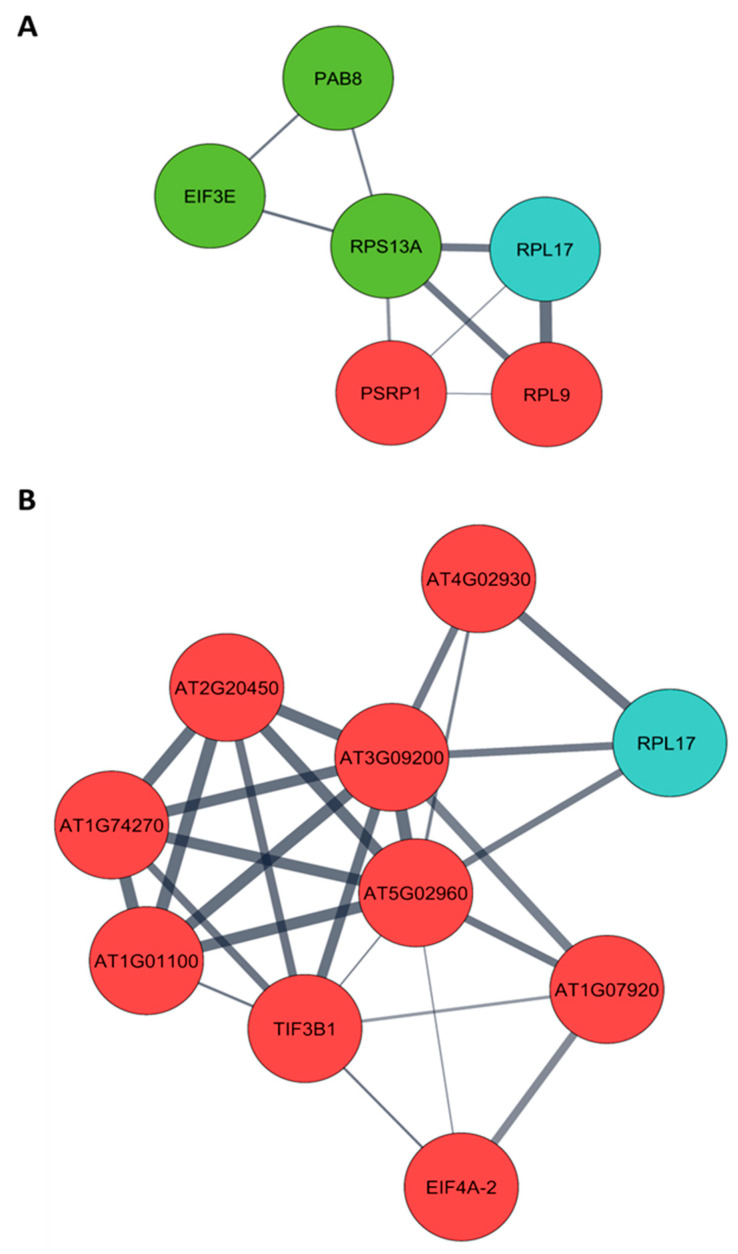
Protein–protein interaction network (PPI) of *Carica papaya* differentially expressed proteins during PMeV complex infection and PMeV CP2 and CP4-interacting proteins. PPI network of PMeV complex-infected *C. papaya* at (**A**) pre-flowering stage (4 months post-germination) and (**B**) post-flowering stage (7 months post-germination). The PPI network was filtered to show proteins involved in translation (see Figure S6 for a complete network). Red nodes are up-regulated proteins; green nodes are down-regulated proteins; blue nodes are PMeV CP2 and CP4-interacting proteins.

Arabidopsis orthologs were obtained from a list of differentially expressed proteins of pre- (4 months post-germination– 4 MPG) and post-flowering (7 months post-germination– 7 MPG) PMeV complex-infected *C. papaya* and submitted to protein–protein interaction (PPI) analysis. From 130 differentially expressed proteins at pre-flowering, it was possible to retrieve 101 orthologs in Arabidopsis (Table S7), while at post-flowering, 123 Arabidopsis proteins were obtained from 160 differentially expressed proteins (Table S8). Two PPI networks were built, including the 28 CP2 and CP4-interacting proteins (Figure 3; Tables S9 and S10). The following CP-interacting proteins were found interacting with several differentially expressed proteins: (i) at 4 MPG, Mediator complex subunit 6 (MED6, AT3G21350), AT2G20270, and AT4G19420; (ii) at 7 MPG, MULTIPLE ORGANELLAR RNA EDITING FACTOR 9 (MORF9, AT1G32580) and ATG22360; (iii) in both networks, NADH Dehydrogenase-like complex L (NdhL, AT1G70760), Flowering locus T (FT, AT1G65480), and ribosomal protein L17 (RPL17, AT3G54210).

### 3.7. CP2 and AtRPL17 Co-Localize and Interact in Nicotiana Benthamiana Cells

**Figure 4:**
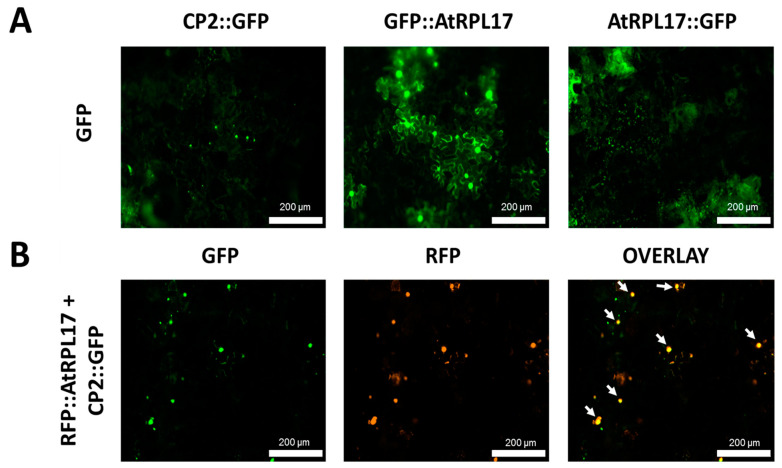
Transient expression and co-localization of *At*RPL17 and CP2 in *Nicotiana benthamiana*. (**A**) Localization of green fluorescent protein (GFP)-fused *At*RPL17 and CP2 proteins (CP2::GFP, *At*RPL17::GFP, and GFP::AtRPL17) in leaf epidermal cells of *N. benthamiana* visualized at 2 days post-infiltration. (**B**) Co-localization of *At*RPL17 and CP2 expressed as fusions to (GFP) or red fluorescent protein (RFP) in *N. benthamiana* epidermal leaf cells. Right column image is GFP and RFP overlayed channels. The fusion proteins RFP::*At*RPL17 were expressed along with GFP::CP2. White arrows: co-localization signals.

**Figure 5:**
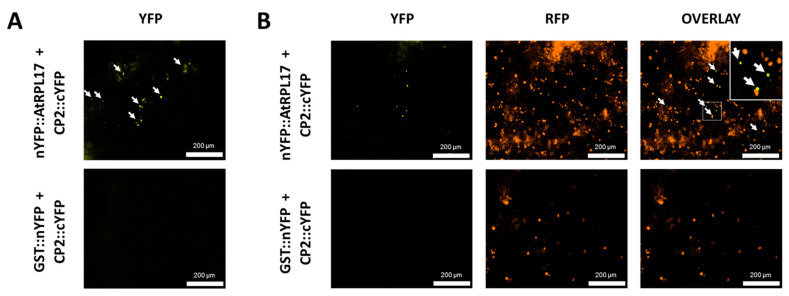
Interaction of *At*RPL17 and CP2 in *Nicotiana benthamiana*. (**A**,**B**) PMeV CP2-*At*RPL17 interaction in vivo by bimolecular fluorescence complementation (BiFC) in wild type (**A**) and transgenic *N. benthamiana* expressing RFP::H2B as a nuclear marker (**B**). Fusion proteins nYFP::*At*RPL17 or GST::nYFP were expressed along with CP2::cYFP by agroinfiltration of the encoding plasmids into leaves of *N. benthamiana*. The reconstitution of yellow fluorescence was visualized 2 days post-infiltration. Fusion protein combinations expressed in each sample are indicated at the left of the corresponding row of images. Inset shows a detailed region of the nuclear marker and the reconstitution of yellow fluorescent protein. White arrows: interaction signals.

To confirm and characterize the interaction of the PMeV capsid protein and *At*RPL17, we first determined the localization of CP2 and *At*RPL17 in plant tissues, fusing GFP to the proteins C- and N-terminus. When GFP was fused to the C-terminus of CP2, punctate fluorescent signals were observed in the epidermal cells of *N. benthamiana* (Figure 4A). No frequent fluorescent signals were detected when GFP was fused to CP2 N-terminus. In parallel, GFP::*At*RPL17 was observed in the nucleus and cytoplasm (Figure 4A), while *At*RPL17::GFP was observed in the cytoplasm only. Co-localization of RFP::*At*RPL17 and CP2::GFP was observed as punctate signals (Figure 4B). BiFC assays showed that both proteins interacted in the cytoplasm, as observed by the agroinfiltration of transgenic *N. benthamiana* expressing RFP fused to the histone protein H2B (Figure 5A,B).

## 4. Discussion

Viruses classified in the order *Ghabrivirales* have been found infecting filamentous fungi, yeast, and protozoa. However, an increasing number of viruses tentatively classified within the order have been found infecting mollusks, arthropods (including mosquitoes, ants, shrimps, and planthoppers), and plants. The knowledge of the interactions of these so-called “toti-like” viruses with their hosts is still limited and much of their features are inferred based on their classified *Totiviridae* member counterparts [33]. Plant-infecting toti-like viruses have been reported [34,35,36,37,38,39,40] (GenBank accessions: NC_009890, ACA61232, MN_119621, ON988291) and despite sharing similar genomic organization, they are phylogenetically distant from papaya meleira virus (PMeV). PMeV is phylogenetically closer to members of the proposed *Fusagraviridae* family, created to accommodate emerging ghabrivirals with similar genomic sequences and an uncharacterized or partially characterized 5′ proximal ORF.

PMeV, the fusagra-like virus, and its umbra-like virus partner, PMeV2, have been implicated with PSD. PSD has been studied at the transcriptome and proteome level, revealing that infected papaya plants present a delay in the appearance of the symptoms until flowering due to a multilayered tolerance mechanism. Additionally, laticifer proteins, cysteine proteases, and a serine protease inhibitor have their accumulation reduced due to PMeV/PMeV2 infection [17,18]. However, key aspects of the virus–host interactions are still unknown, hindering the development of more effective strategies for PSD control. In this scenario, viral capsid proteins are an important target of study as they possess a multifunctional nature beyond protecting the viral genome [11,12,13,14].

Here we characterized the structural protein coding region of PMeV, a candidate member of the proposed *Fusagraviridae* family. PMeV virion contents separate into two major proteins, p40 and p55, and p55 is the PMeV ORF1 translation product processed or degraded at position 263. In addition, a fragment of p55 (CP2, aa 321–670) interacts with a viral structural protein fragment, CP4 (aa 961–1200), and they both interact with 28 plant proteins mostly targeted to the chloroplast. One of these proteins, the 50S ribosomal protein L17 (RPL17), co-localized and interacted with CP2 in *N. benthamiana* cells and is associated with translation-related proteins differentially modulated in pre-flowering *C. papaya*.

The first analysis to identify the nature of the PMeV capsid protein applied trypsin digestion to the crude sucrose-purified virions followed by mass spectrometry analysis (MS/MS), identifying peptides matching with the deduced PMeV ORF1 protein sequence [3]. The additional cesium chloride isopycnic purification followed by SDS-PAGE led us to identify the structural proteins of PMeV virions. Particles from M and B fractions presented the same separation profile on SDS-PAGE, giving additional support to the transcapsidation between PMeV and PMeV2 (Figure 1C). Through LC-MS/MS of the purified virions, 100 unique peptides were identified matching two main regions of the protein, one spanning amino acids 283 to 739 and another spanning amino acid 880 to 1451, leaving an in-between gap of 140 amino acids (~15 kDa) with no peptides identified.

Interestingly, no peptides were identified in the N-terminal end of the predicted protein. A lack of the predicted N-terminal region in the ORF1-encoded protein has been noted for the fusagravirus cryphonectria carpinicola fusagravirus 1 (CcFGV1) [41], and the ghabrivirals yadonushi virus 1 (YkV1) (unclassified ghabriviral) [10] and infectious myonecrosis virus (IMNV) (tentatively classified in the *Totiviridae* family) [42]. The sequencing of the N-terminal end of the ~55 kDa band obtained from cesium chloride-purified virions mapped the first peptide at position 263, which supports the idea that this region was not detected by mass spectrometry or may not be present in the mature capsids.

Based on our results, we propose that a protein of ~55 kDa (p55) spanning amino acids (263–739) is one of the structural proteins of the PMeV capsid. A model predicted by AlphaFold revealed a superimposed structure shared between p55 and the conserved domain of the capsid proteins of dsRNA fungal viruses in the families *Chrysoviridae* and *Totiviridae*, supporting the idea of p55 as the PMeV major capsid protein. The identification of peptides after the gap indicates that a protein derived from the C-terminal half of the ORF1-product (880–1563, ~77 kDa) is also part of the PMeV virion (possibly p40). Proteins of ~100 kDa, ~85 kDa, ~68 kDa, ~40 kDa, and lower than 40 kDa are visualized in our SDS-PAGE, but it remains to be determined whether they are the result of further processing or degradation of a primary translation product, or the result of an alternative translation strategy.

The production of small proteins from a larger predicted protein is a very common phenomenon within RNA viruses. This can be achieved either by the initiation of translation from internal ribosomal entry sites (IRES), by ribosomal skipping, frameshifting, termination-reinitiation, leaky scanning, shunting, or by programmed cleavage of a larger protein (by virus or host proteases) (reviewed in [43,44]). Sites for ribosomal frameshifting are found among toti-like viruses and it is the assumed expression strategy for its RdRp. Ribosomal skipping is seen among the toti-like viruses of the proposed Artivirus clade, which possess 2A-like sequences in their genomes which mediate a skipping effect of the ribosome resulting in an apparent co-translational cleavage of proteins, therefore lacking the need for a protease. Pseudo 2A-sites are found in the Giardiavirus clade, but their amino acid composition makes them unlikely to produce a skipping effect [27]. No 2A-like sequences or pseudo 2A-sites are found in the PMeV ORF1, which suggests another strategy for ORF1-encoded protein processing.

It is noteworthy that the PMeV complex is, hitherto, the only virus described to inhabit *C. papaya* laticifer cells, a physical and chemical defense barrier against pathogens. Laticifers are the only cells where the PMeV complex is found [1] and it is during the laticifer differentiation that papaya proteases are accumulated. Early laticifer cells undergo autophagy of their well-developed organelles, but later in differentiation, their endoplasmic reticulum splits into fragments and initiates the production and accumulation of proteases which are stored within vesicles of the mature laticifer [4,45]. Several viruses encode proteases which are necessary for post-translational modifications of their proteins to ensure that proteins can travel together to the viral assembly site, to ensure the proper timing for the initiation of folding and assembly, and to control the concentration of key viral proteins [44]. However, viruses that do not encode a protease rely on their host counterparts for protein processing, as seen with two members of the *Totiviridae* family. A specific host cysteine protease separates the capsid and replicase polyprotein of Giardiavirus (GLV) and Leishmania RNA virus (LRV) [46,47]. In the prototype of the genus *Victorvirus* (family *Totiviridae*), helminthosporium victoriae virus 190S (HvV190S), proteolytic processing mediated by unknown proteases results in HvV190S structural proteins. Only one ORF is predicted for HvV190S, but SDS-PAGE of purified virions shows three forms of the CP, p88, p83, and p78, named after their relative molecular masses [48]. Interestingly, p83 and p78 are products of the proteolytic processing of p88, although no protease-like protein is coded by HvV190S [49]. Here, we identified 28 Arabidopsis proteins interacting with two fragments of PMeV ORF1-encoded protein. One of these proteins, a cysteine protease, has homologs identified in PMeV complex-infected *C. papaya* green tissues and latex [17,18,19]. In this work, LC-MS/MS on viral preparations from infected *C. papaya* latex identified three cysteine proteases that co-purify with PMeV complex virions and are 33% identical to the Arabidopsis homologous proteins. This supports the idea that cysteine proteases are essential players during the PMeV complex infection of *C. papaya* and might be responsible for processing the ORF1 product. Alternatively, a serine protease activity could result in p55 production, as sites for serine proteases were seen in both N- and C-terminal ends of p55. Quantitative analysis on the latex of disease plants shows a reduction in the accumulation of a serine protease inhibitor (UniProt accession: P80691) [17,18], which might represent a viral strategy to tune protein processing. Alternatively, a serine protease activity of the PMeV ORF1 product cannot be discarded, as well as the role of PMeV2 still-uncharacterized proteins.

Using Y2H, no dimerization or expression was detected for the full-length ORF1. Besides yeast, the full-length ORF1 translation product is undetectable when expressed in *N. benthamiana*, *Spodoptera frugiperda* Sf9 insect cells, and *E. coli*, indicating that a particular environment is necessary for its correct expression and processing. Indeed, using transmission electron microscopy, Kitajima et al. [1] analyzed different tissues and organs of PMeV complex-infected *C. papaya* looking for virions, but they were not observed in any other tissue besides the laticifers. *C. papaya* latex is composed of lipids, phenols, alkaloids, sugars, oxalate crystals, polyisoprenoids, and mostly proteins [9]. Proteins identified in latex [26,27] and their accumulation level associated with the physiological milieu of laticifer cells might be important for the correct translation and processing of PMeV ORF1, as well as its interaction with cellular factors.

The dimerization assay using the yeast two-hybrid system revealed that CP4 homodimerizes and interacts with the other three fragments of PMeV ORF1-encoded protein, CP2 (region within p55), and CP3, but strongly with CP2. We confirmed that CP4 is part of the assembled capsid as 17 unique peptides were found in the LC-MS/MS from purified virions matching with the region spanning CP4. If not p40, CP4 can be part of the minor proteins visualized in our SDS-PAGE. Minor bands with a relative molecular weight lower than the major capsid protein (20–40 kDa) are seen for infectious myonecrosis virus (IMNV, tentatively classified in the *Totiviridae* family), and they have been pointed as forming fiber complexes protruding from the capsid, probably mediating transmission and infection processes [50]. CP2/p55 interaction with CP4 supports the idea that these segments interact to form units of the assembled capsid. Thus, it is reasonable to suggest that CP4 is part of the assembled capsid and might play an important role during the virus life cycle as it interacts with several plant proteins.

The yeast two-hybrid screening with the Arabidopsis library identified 28 proteins interacting with CP2 and CP4. To choose a protein that could play an important role in the PMeV complex-*C. papaya* pathosystem, we built a PPI network using Arabidopsis orthologs of *C. papaya* proteins modulated during PMeV infection at 4- and 7-MPG. The 50S ribosomal protein L17 (RPL17) appeared in both scenarios as putatively associated with four up-regulated proteins and one down-regulated protein at 4 MPG, and with three up-regulated proteins and one down-regulated protein at 7 MPG. It is interesting to note that at 4 MPG, RPL17 is associated with several down-regulated proteins related to protein synthesis and ribosome biogenesis, which includes ribosomal protein S13A (RPS13A, AT4G00100), eukaryotic translation initiation factor 3E (EIF3E, AT3G57290), poly(A) binding protein 2 (PAB8, AT1G49760), fibrillarin 2 (FIB2, AT4G25630), and NOP56-like pre-RNA processing ribonucleoprotein (NOP56-like, AT3G05060). In addition, translation complexes are found co-purifying with PMeV virions, which include proteins involved in the regulation of translation initiation, such as eukaryotic translation initiation factor 4A (A0A0P0INT0), eukaryotic translation initiation factor 2c (A0A072VPN5), an MI domain-containing protein (A0A0D3BWP8), and proteins involved in mRNA capping such as mRNA (guanine-N(7))-methyltransferase (A0A5B8MBX6) and nuclear cap-binding protein subunit 1 (A0A438F2I9), which may be usurped by the virus for its protein synthesis.

Ribosomal proteins have been reported to directly affect several processes during virus infections, either with a pro- or antiviral activity [51,52]. A proviral function has been observed in the translational transactivation of cauliflower mosaic virus (CaMV), in which RPL18 of *A. thaliana* interacts with P6 of CaMV in a complex comprised of RPs including L18, L24, and eIF3 [53]. On the other hand, it has been shown that RPL10 is an important player in the antiviral defense pathway in plants. The phosphorylation of RPL10 by its specific partner, the geminiviral nuclear shuttle protein-interacting kinase, redirects it to the nucleus to modulate viral infection [54]. The fact that translation-regulating proteins are down-accumulated at 4 MPG gives support to the tolerance mechanism presented by *C. papaya* at pre-flowering, and the interaction of RPL17 with the PMeV complex capsid protein could be detrimental to this process.

Although modulation of translation could be an important mechanism used by the PMeV complex or its host to regulate virus levels, several other cellular mechanisms could be altered due to the interaction of plant proteins with the PMeV capsid protein. Our results point to other processes, including polyprotein and RNA processing, cell wall modification, gene expression regulation, and reactive oxygen species detoxification that have been identified as modulated in a previous transcriptome [20] and proteome [19] analysis of PMeV complex-infected *C. papaya*. Thus, identifying binding partners of PMeV CP provides a framework for a better understanding of the response of plants against PMeV and identifies new targets for developing more effective strategies to control PSD.

## Figures and Tables

**Table 1:** Yeast two-hybrid-derived clones obtained from a screening using PMeV ORF1 fragment 4 (CP4) as bait.

Clone No.	Growth Media	NCBI or TAIR Description	TAIR Accession	Gene Ontology Information
1	QXA	Beta-1,4-N-acetylglucosaminyltransferase family protein	AT3G01620	Located in Golgi apparatus
2		Zinc finger protein 2	AT5G57520	Located in nucleus
3		DHHC-type zinc finger family protein	AT2G40990	Is active in Golgi apparatus and endoplasmic reticulum
4		Polynucleotide adenylyltransferase family protein	AT5G23690	Involved in RNA processing
5		Transmembrane protein	AT2G35750	Located in mitochondrion
6		Inorganic carbon transport protein-like protein (NdhL)	AT1G70760.1	Located in chloroplast, chloroplast thylakoid membrane, thylakoid membrane
7	QXA 2.5 mM	Cytochrome c oxidase assembly protein CtaG / Cox11 family	AT1G02410	Located in chloroplast, integral component of mitochondrial membrane, mitochondrion
8		Pectin lyase-like superfamily protein	AT1G04680	Located in extracellular region
9		Plastid developmental protein DAG (MORF9)	AT1G11430	Located in chloroplast, chloroplast envelope, chloroplast stroma
10		Plant/protein	AT1G13990	Located in chloroplast
11		Peroxidase CB (PRXCB)	AT3G49120	Located in Golgi apparatus, apoplast, cell wall, cytosol, extracellular region, plant-type cell wall, plant-type vacuole, secretory vesicle
12		RmlC-like cupins superfamily protein	AT1G03890	Located in extracellular region
13		RNA polymerase transcriptional regulation mediator-like protein (MED6)	AT3G21350	Located in nucleus
14		Chloroplast ribosomal protein S3	ATCG00800.1	Located in chloroplast, chloroplast envelope, chloroplast nucleoid, chloroplast stroma, plastid
15		Chloroplast GRX 12, GRXS12	AT2G20270	Located in chloroplast, chloroplast stroma, mitochondrion
16	QXA 5 mM	DNAJ heat shock family protein	AT2G22360	Located in chloroplast, chloroplast envelope, chloroplast thylakoid membrane, cytoplasm, vacuole
17		Ribosomal protein L17 family protein	AT3G54210	Located in chloroplast, chloroplast envelope, chloroplast stroma, cytosol
18		Sec14p-like phosphatidylinositol transfer family protein	AT1G72160	Located in plasma membrane
19		GDSL-like Lipase/Acylhydrolase superfamily protein	AT5G45670	Located in extracellular region
20		Chaperone protein dnaJ-like protein	AT5G06130	Located in chloroplast membrane, mitochondrion
21		GPI-anchored protein	AT3G18050	Located in anchored component of membrane, chloroplast
22		Pyrimidin 4 (PYR4)	AT4G22930	Located in chloroplast, cytosol, mitochondrion
23		Pectinacetylesterase family protein	AT4G19420	Located in extracellular region
24		Double Clp-N motif protein	AT4G12060	Located in chloroplast, chloroplast envelope, chloroplast stroma, cytosol, plastid stroma
25		PEBP (phosphatidylethanolamine-binding protein) family protein (FT)	AT1G65480	Located in cytoplasm nucleus
26		Clone RAFL09-89-G08 (R19778) putative cellulose synthase catalytic subunit (RSW1)	AT4G32410.1	Located in Golgi apparatus, endosome, plasma membrane, trans-Golgi network
27		mRNA for plastid protein, complete cds, clone: RAFL15-06-D14	AT1G32580.1	Located in chloroplast, mitochondrion, nucleus
28		Papain family cysteine protease	AT4G16190	Located in extracellular region, lytic vacuole, plant-type vacuole, vacuole

## Data Availability

Not applicable.

## Supplementary Materials

The following supporting information can be downloaded at: https://www.mdpi.com/article/10.3390/v15020541/s1, Figure S1: N-terminal sequencing of 55kDa band. Each figure displays the chromatographic traces for each cycle ordered from N- to C- terminal. Figure S2: Three-dimensional structure of PMeV p55 using AlphaFold. (A) Ribbon diagram of p55 (diagram was positioned for easy visualization of secondary structures) rainbow-colored from blue (N-terminus) to red (C-terminus). (B) Sequence of p55 and its respective secondary structures. The α-helices (rectangles) and β-strands (arrows) are rainbow-colored from blue (N-terminus) to red (C-terminus); Figure S3: Structural alignment of p55 and PcV capsid protein (CP) domain A. (A) Sequence alignment of p55 and PcV CP domain A resulting from the Dali structural alignment. The α-helices (rectangles) and β-strands (arrows) are rainbow-colored from blue (N-terminus) to red (C-terminus) for each protein. Conserved residues are represented in red (B) Superimposed structures of p55 (yellow) and PcV CP domain A (blue). White regions indicate non-superimposed regions for both domains. Figure S4: Superimposed structures of p55 (yellow), Penicillium chrysogenum virus (blue), Saccharomyces cerevisiae virus L-A (white), Omono river virus (green) capsid proteins. Figure S5: Spot plating shows the validation of genuine positives interacting with CP2 and CP4. Figure S6: Protein–protein interaction network (PPI) of *C. papaya* differentially accumulated proteins during papaya meleira virus (PMeV) complex infection and PMeV CP2 and CP4-interacting proteins; Table S1: List of primers used to generate the clones in this study; Table S2: LC-MS/MS analysis of tryptic peptides obtained from PMeV complex virions; Table S3: Dali-based structural comparisons of PMeV p55 against protein structure database; Table S4: Metrics of the LC-MS/MS analysis of plant proteins co-purifying with PMeV complex virions; Table S5: LC-MS/MS analysis of host plant proteins co-purifying with PMeV complex virions; Table S6: Similarity of CP2 and CP4 Arabidopsis protein with *C. papaya* proteins; Table S7: Arabidopsis orthologs obtained from proteins differentially accumulated in infected papaya leaf at 4 months post germination; Table S8: Arabidopsis orthologs obtained from proteins differentially accumulated in infected papaya leaf at 7 months post germination. Table S9: Arabidopsis orthologs obtained from proteins differentially accumulated in infected papaya leaf (at 4 months post germination) used to retrieve the protein–protein interaction network of CP2 and CP4-interacting proteins; Table S10: Arabidopsis orthologs obtained from proteins differentially accumulated in infected papaya leaf (at 7 months post germination) used to retrieve the protein–protein interaction network of CP2 and CP4-interacting proteins.
