# Supplementary material for: A Capsid Protein Fragment of a Fusagra-like Virus Found in Carica papaya Latex Interacts with the 50S Ribosomal Protein L17
Source: Viruses. 2023 Feb 15;15(2):541. doi: 10.3390/v15020541 (PMC9961563; doi:10.3390/v15020541)
Supplement: Supplementary file 1 [file viruses-15-00541-s001.zip › Supplementary data.pdf]

Supplementary data

A capsid protein fragment of a fusagra-like virus found in *Carica papaya* latex interacts with the 50S ribosomal protein L17

Marlonni Maurastoni[1,8], Tathiana F. Sá-Antunes[1], Emanuel F. M. Abreu[2], Simone G. Ribeiro[2], Angela Mehta[2], Marcio M. Sanches[2], Wagner Fontes[3], Elliot W. Kitajima[4], Fabiano T. Cruz[1], Alexandre M. C. Santos[1], Jose A. Ventura[1,5], Ana C. M. M. Gomes[2], Francisco M. Zerbini[6], Patricia Sosa-Acosta[7], Fábio C. S. Nogueira[7], Silas P. Rodrigues[8], Francisco J. L. Aragão[2], Anna E. Whitfield[9] and Patricia M. B. Fernandes[1*]

[1] Biotechnology Core, Federal University of Espírito Santo, Vitória, ES 29043-900, Brazil
[2] Embrapa Recursos Genéticos e Biotecnologia, Brasília, DF 70770-917, Brazil
[3] Department of Cell Biology, University of Brasilia, Brasilia, DF 70910-900, Brazil
[4] Department of Phytopathology, University of São Paulo, Piracicaba, SP 13418-900, Brazil
[5] Espírito Santo Institute for Research, Technical Assistance and Rural Extension, Vitória, ES 29052-010, Brazil
[6] Department of Phytopathology, Federal University of Viçosa, Viçosa, MG 36570-900, Brazil
[7] Department of Biochemistry, Institute of Chemistry, Federal University of Rio de Janeiro, Rio de Janeiro, RJ 21941-909, Brazil
[8] Multidisciplinary Core for Research in Biology, Campus Duque de Caxias, Federal University of Rio de Janeiro, Duque de Caxias, RJ 25240-005, Brazil
[9] Department of Entomology and Plant Pathology, North Carolina State University, 840 Main Campus Drive, Raleigh, NC 27606, USA
* Correspondence: patricia.fernandes@ufes.br

SAMPLE (pink peaks) and STANDARD (grey peaks)

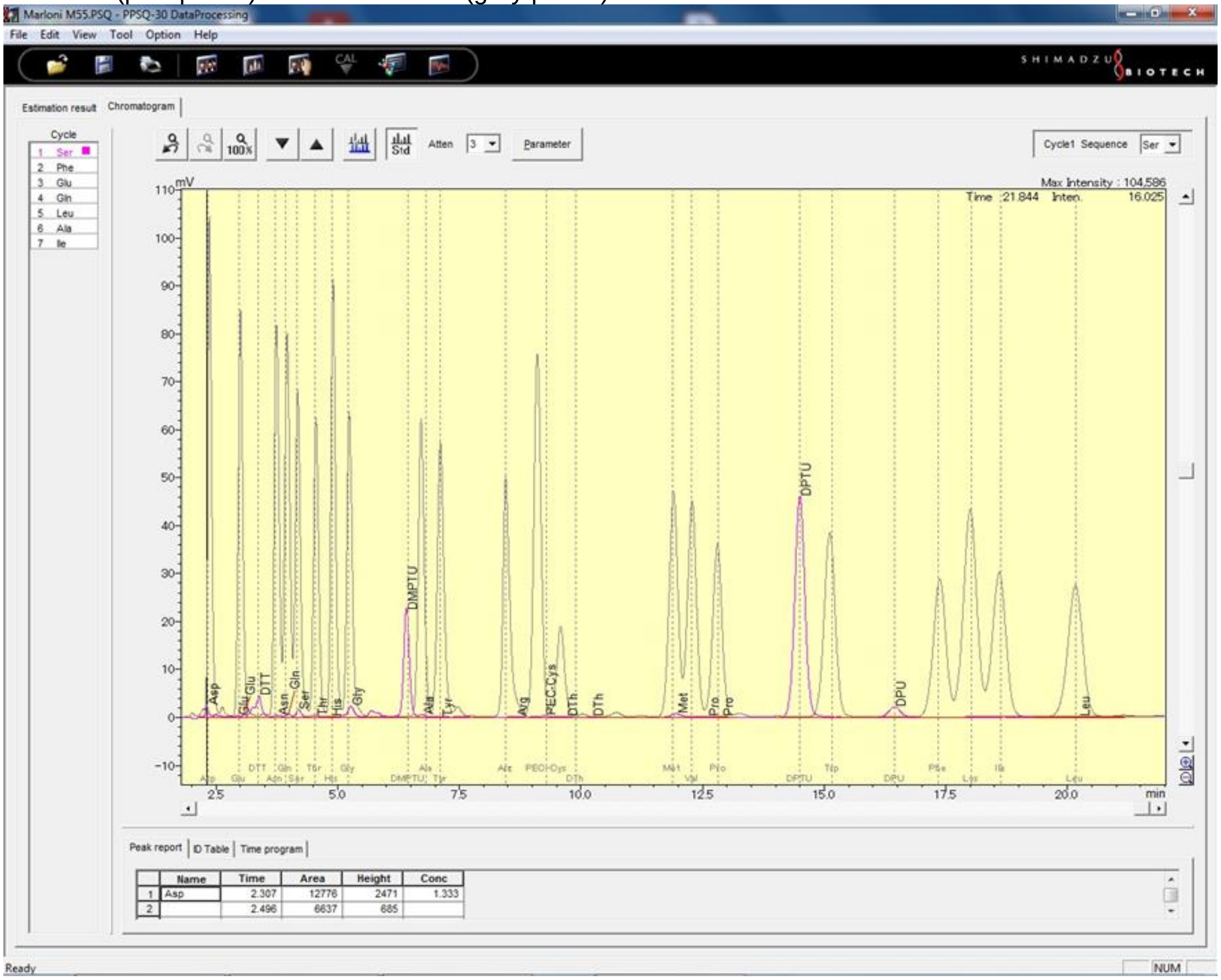

SERINE (S) / GLYCINE (G)

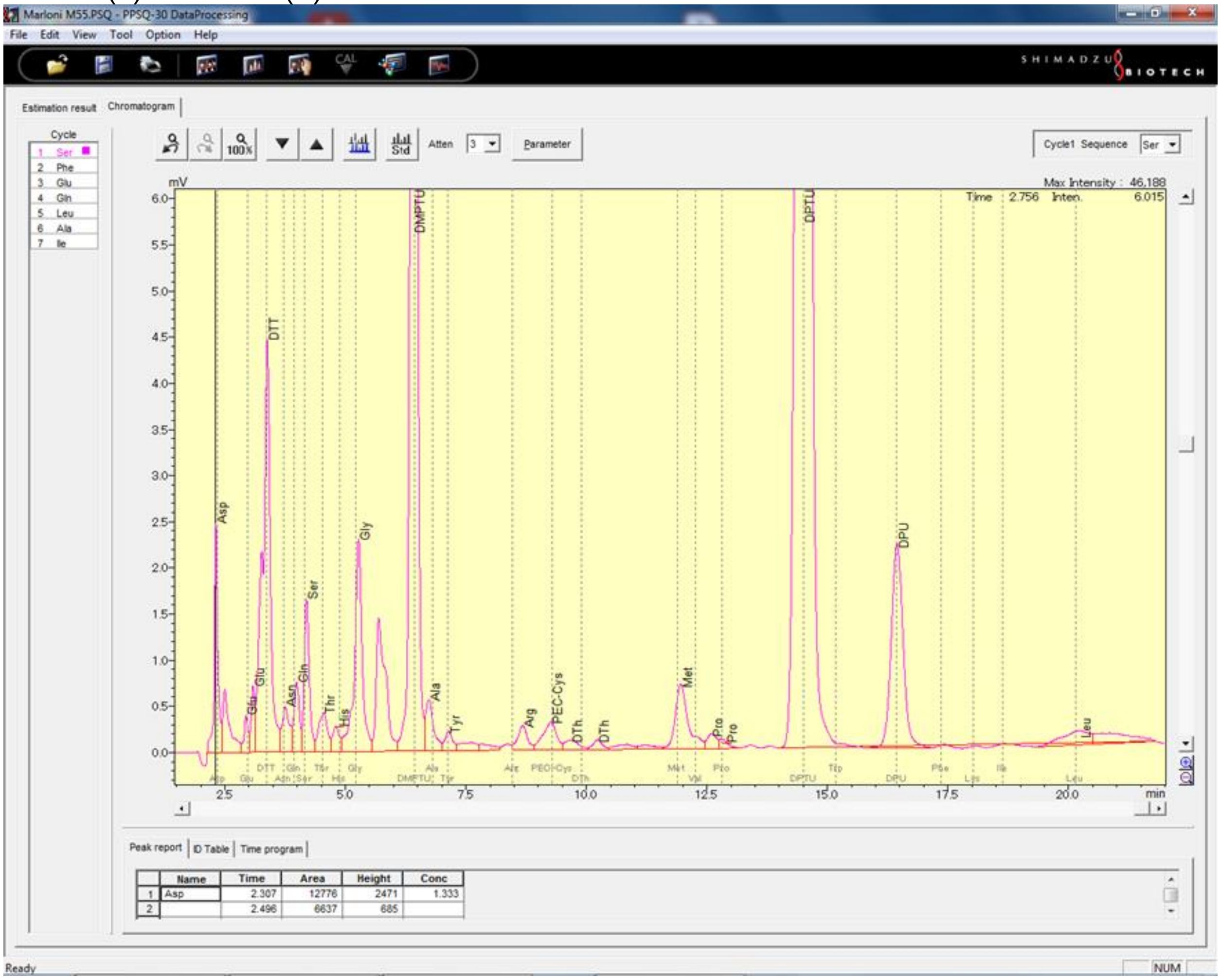

ANY AMINO ACID X

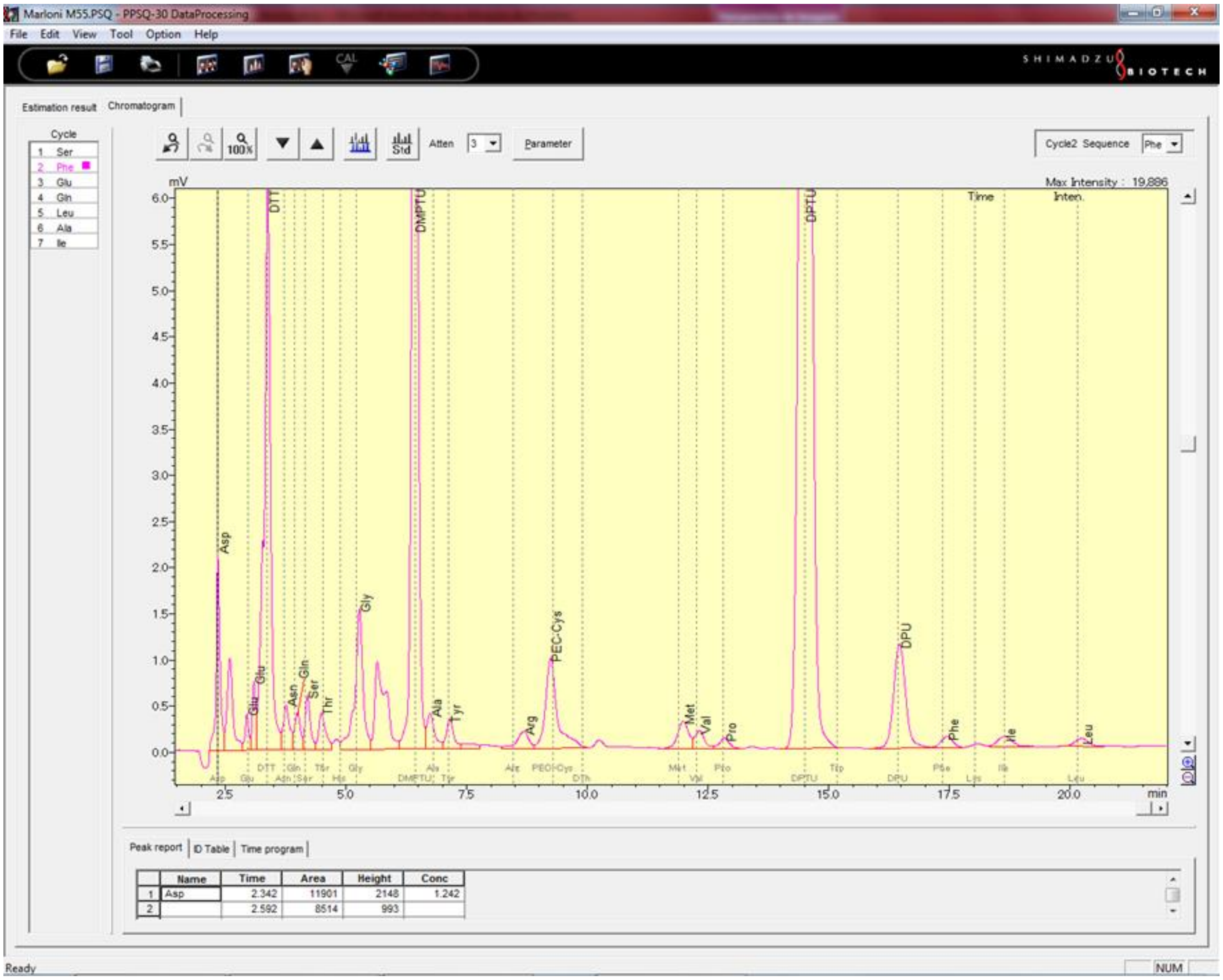

	Name	Time	Area	Height	Conc
1	Asp	2.342	11901	2148	1.242
2		2.592	8514	993	

GLUTAMIC ACID E

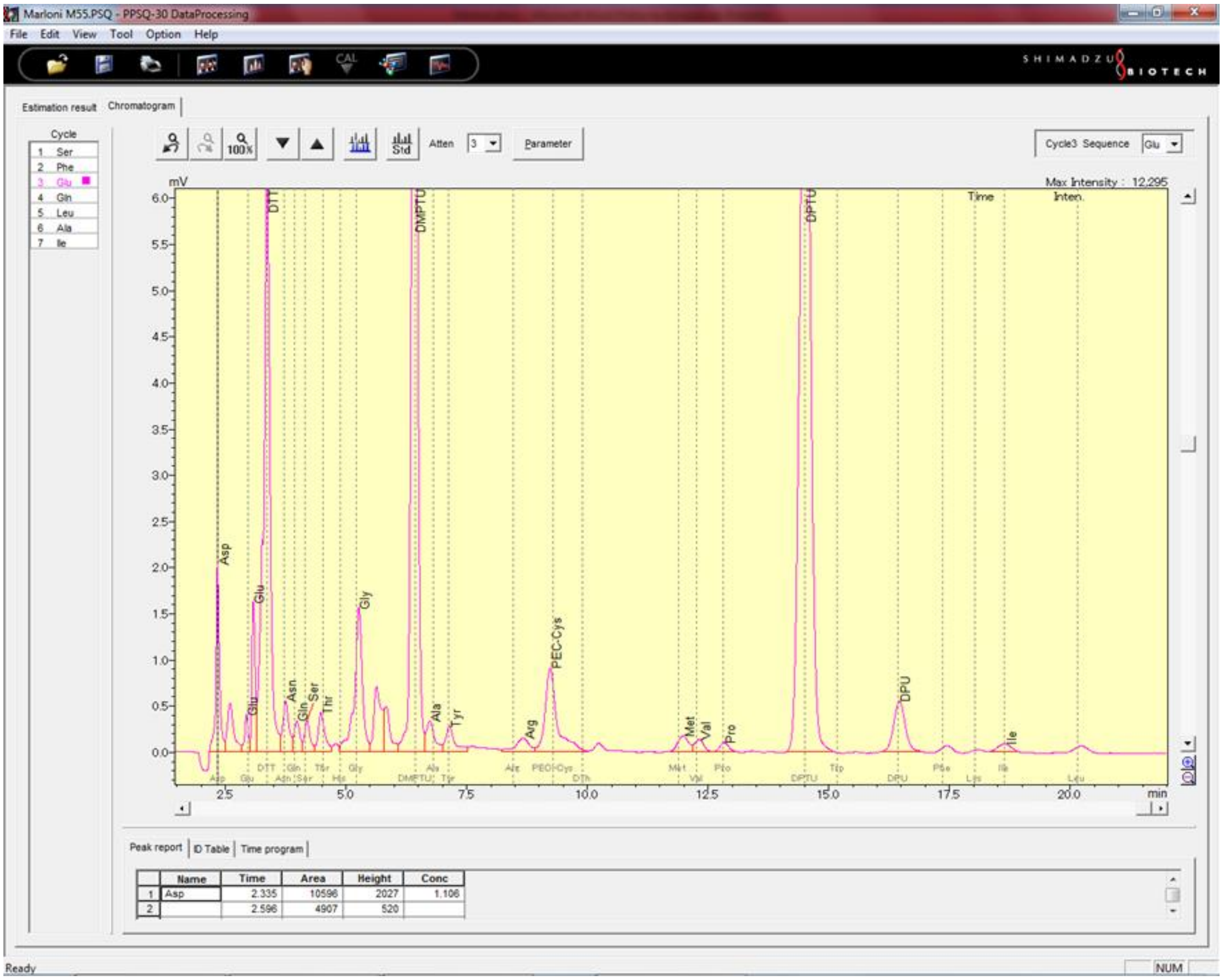

PHENYLALANINE F

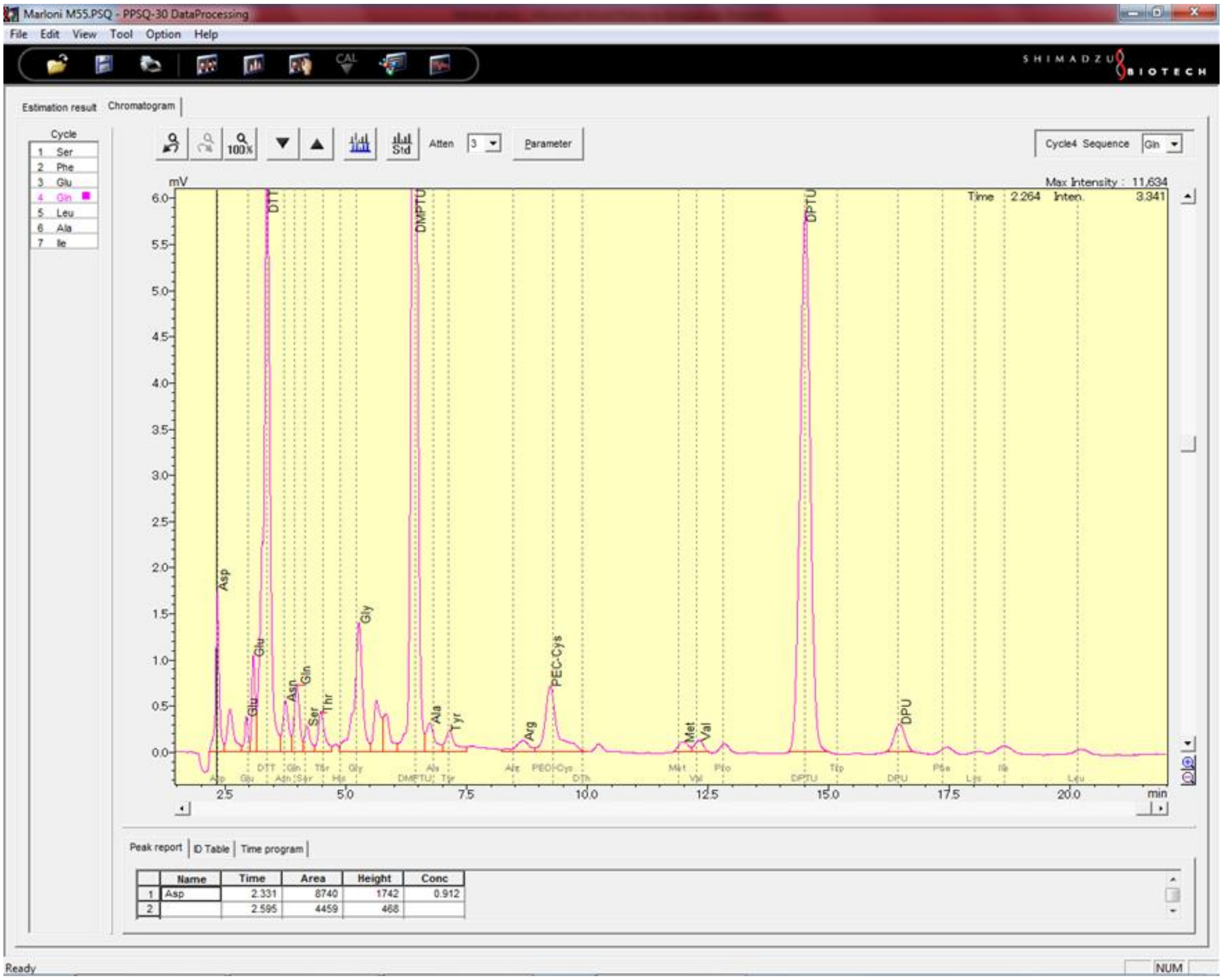

LEUCINE L

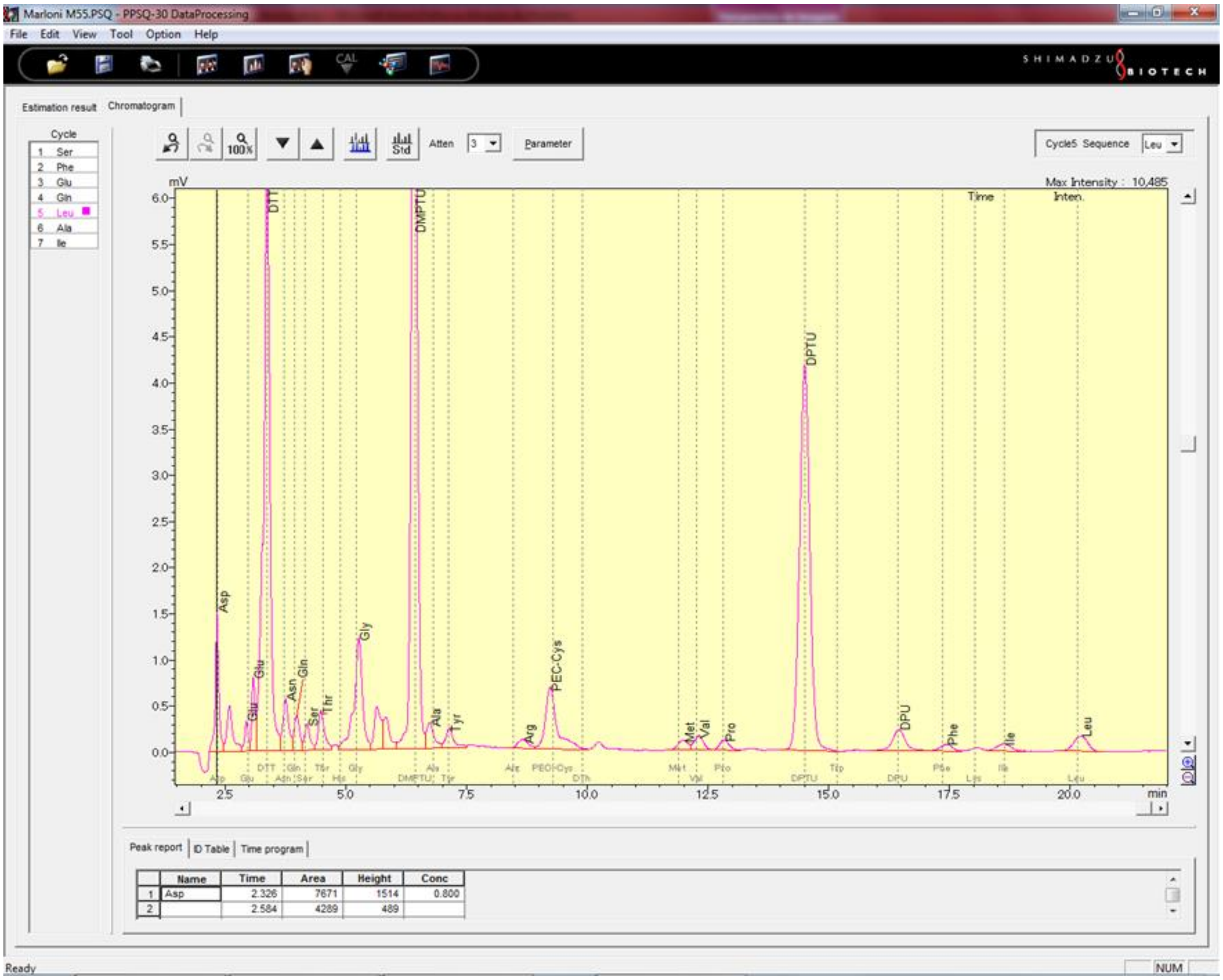

Marloni M55.PSQ - PPSQ-30 DataProcessing
File Edit View Tool Option Help
SHIMADZU BIOTECH
Estimation result
Chromatogram
Cycle
1 Ser
2 Phe
3 Glu
4 Gln
5 Leu
6 Ala
7 Ile
Atten 3
Parameter
Cycle5 Sequence Leu
mV
Max Intensity : 10,485
Time
Inten.
Asp
Glu
DTT
Asn
Gln
Ser
Thr
Gly
DMPTU
Ala
Tyr
Arg
PEC-Cys
Met
Val
Pro
DPTU
DPU
Phe
Ile
Leu
min
Peak report
ID Table
Time program
Name | Time | Area | Height | Conc
1 Asp | 2.326 | 7671 | 1514 | 0.800
2 | 2.584 | 4289 | 489 |
Ready
NUM

ALANINE (A)

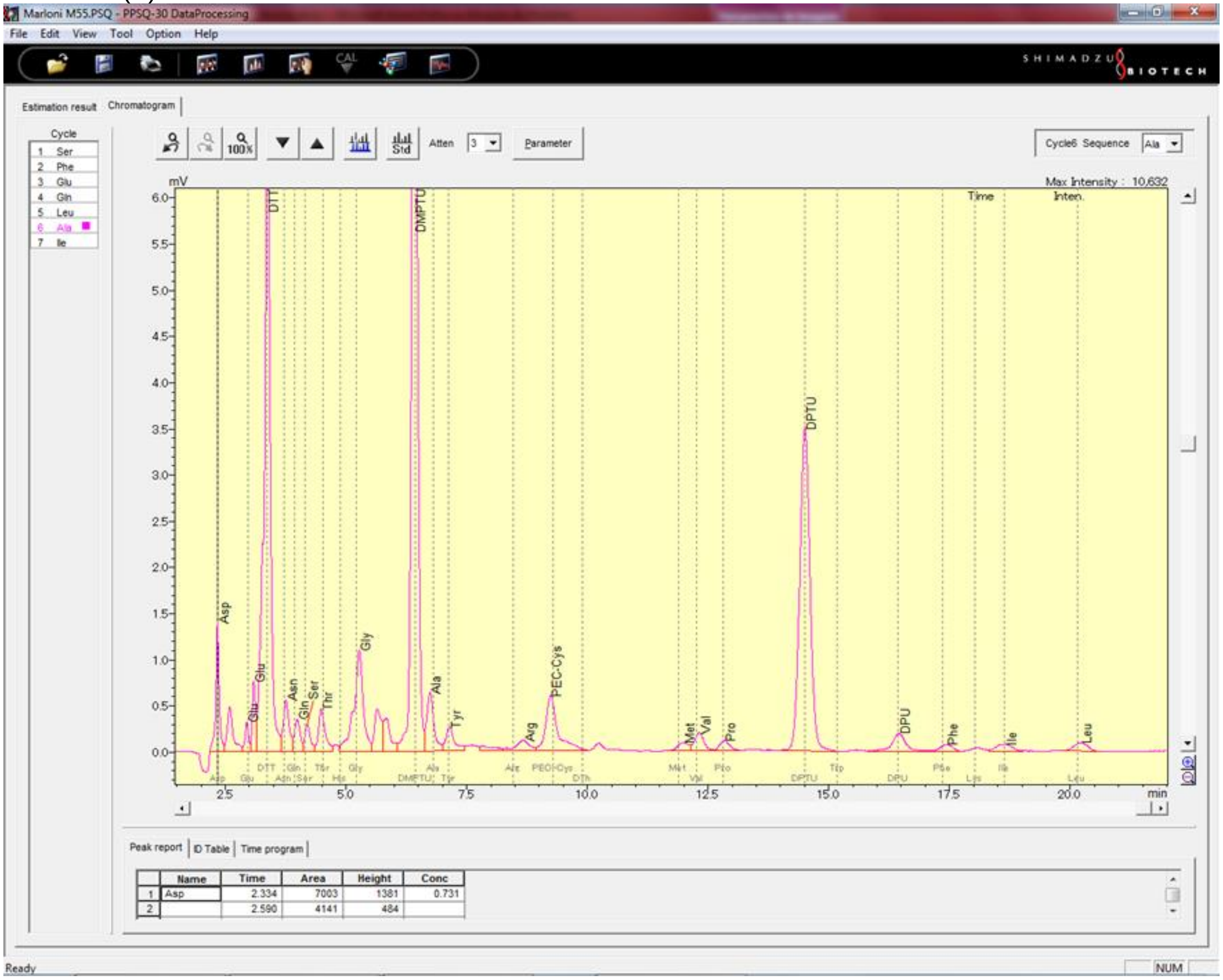

Marloni M55.PSQ - PPSQ-30 DataProcessing
File Edit View Tool Option Help
SHIMADZU BIOTECH
Estimation result
Chromatogram
Cycle
1 Ser
2 Phe
3 Glu
4 Gln
5 Leu
6 Ala
7 Ile
Atten
Parameter
Cycle6 Sequence Ala
mV
Max Intensity : 10,632
Time
Inten.
Asp
Glu
DTT
Asn
Gln
Ser
Thr
Gly
DMPTU
Ala
Tyr
Arg
PEC-Cys
Met
Val
Pro
DPTU
DPU
Phe
Ile
Leu
min
Peak report
ID Table
Time program
Name
Time
Area
Height
Conc
1 Asp 2.334 7003 1381 0.731
2 2.590 4141 484
Ready
NUM

ISOLEUCINE I

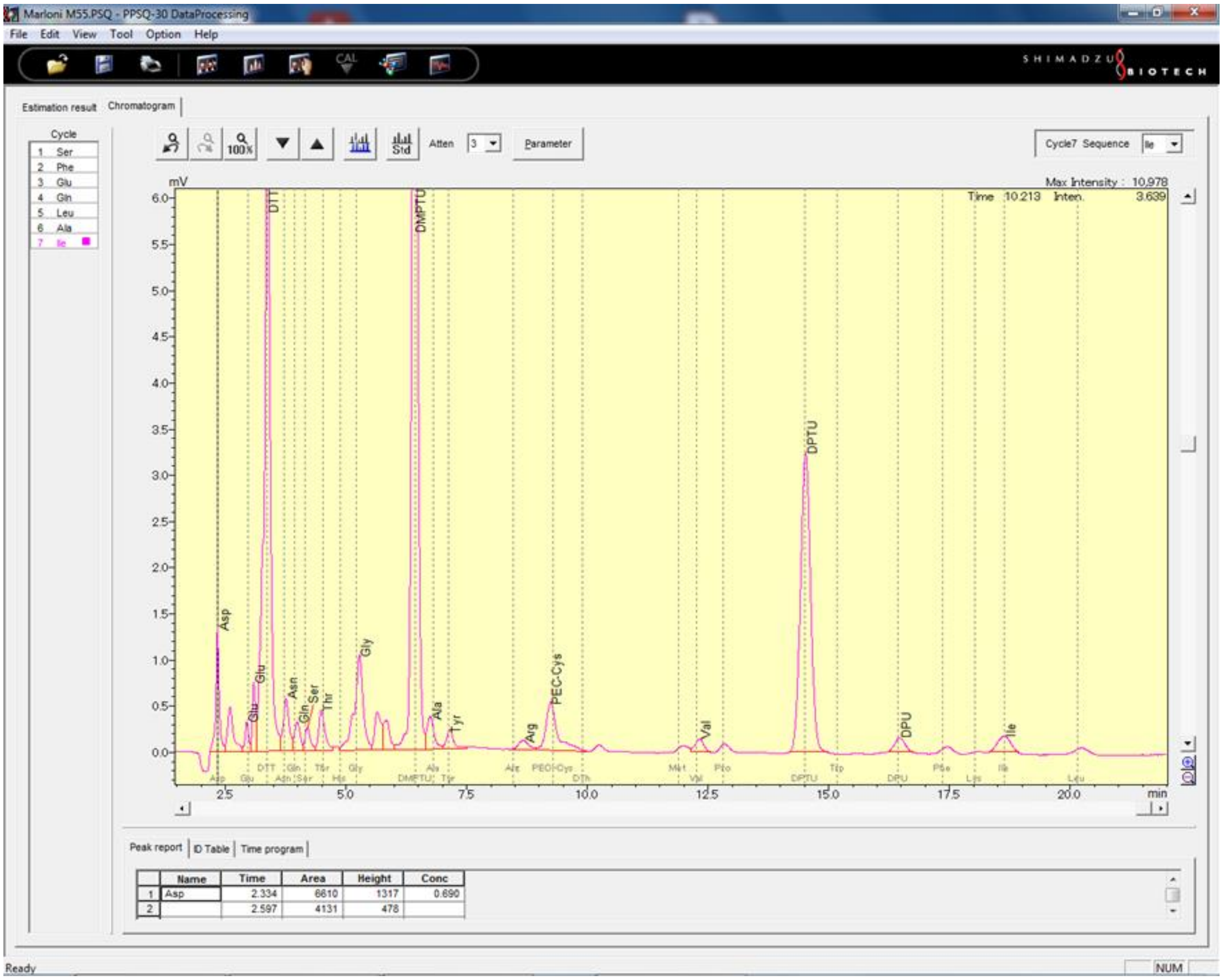

Supplementary Figure S1. N-terminal sequencing of 55kDa band. Each figure displays the chromatographic traces for each cycle ordered from N- to C- terminal.

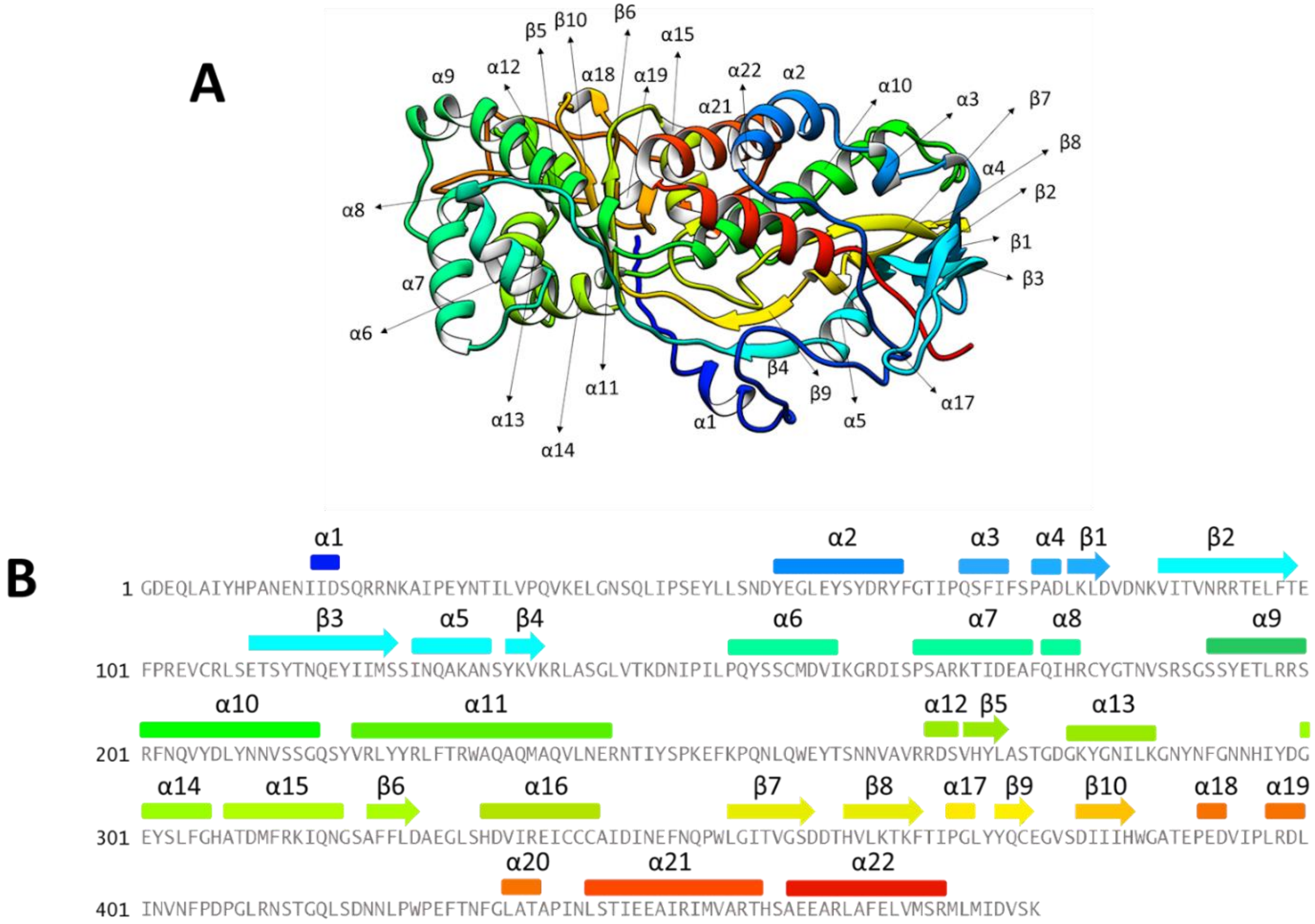

Supplementary Figure S2. Three-dimensional structure of PMeV p55 using AlphaFold. **A.** Ribbon diagram of p55 (diagram was positioned for easy visualization of secondary structures) rainbow-colored from blue (N-terminus) to red (C-terminus). **B.** Sequence of p55 and its respective secondary structures. The α-helices (rectangles) and β-strands (arrows) are rainbow-colored from blue (N-terminus) to red (C-terminus).

A

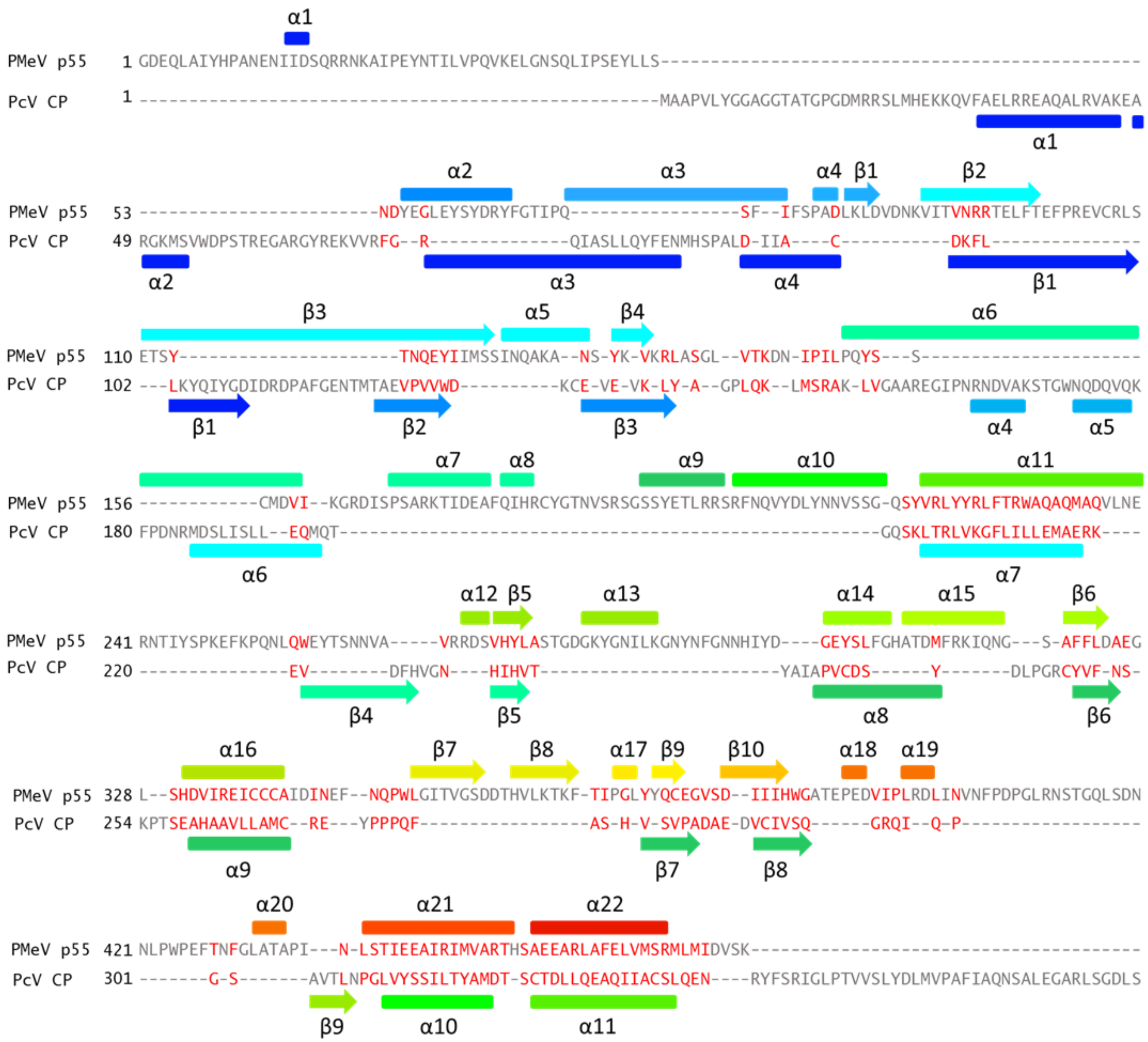

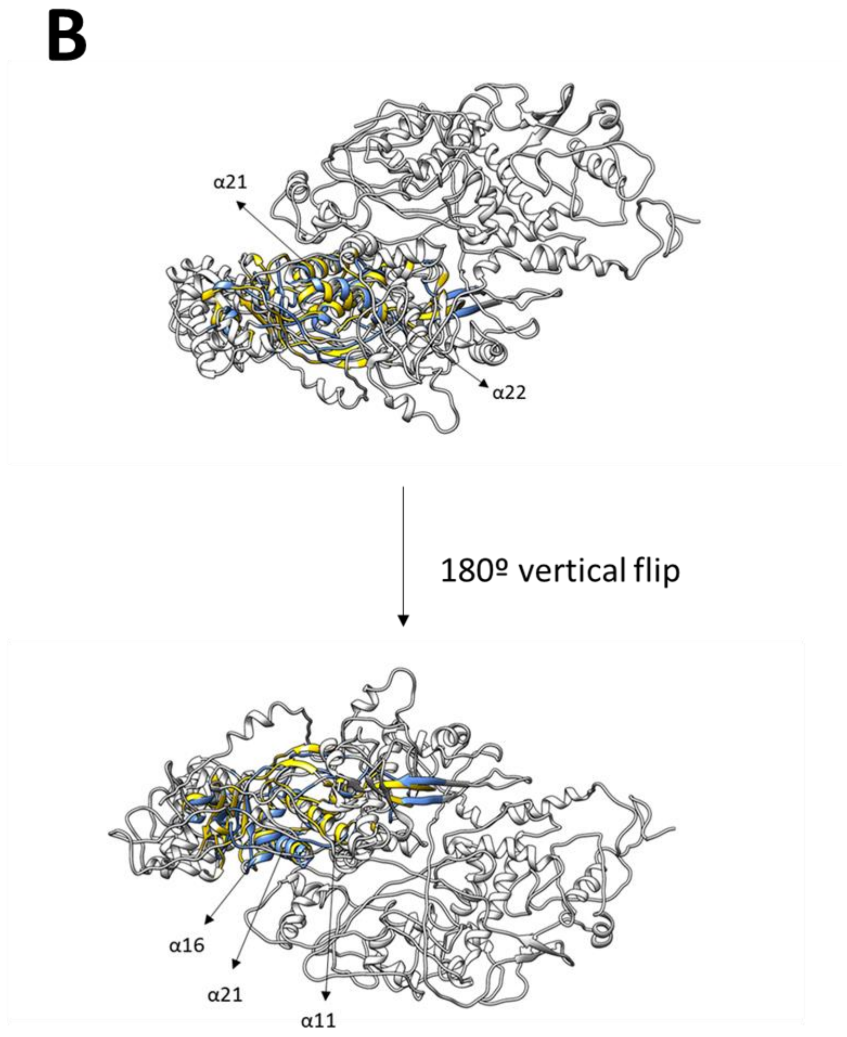

Supplementary Figure S3. Structural alignment of p55 and PcV capsid protein (CP) domain A. **A.** Sequence alignment of p55 and PcV CP domain A resulting from the Dali structural alignment. The α-helices (rectangles) and β-strands (arrows) are rainbow-colored from blue (N-terminus) to red (C-terminus) for each protein. Conserved residues are represented in red. **B.** Superimposed structures of p55 (yellow) and PcV CP domain A (blue). White regions indicate non superimposed regions for both domains.

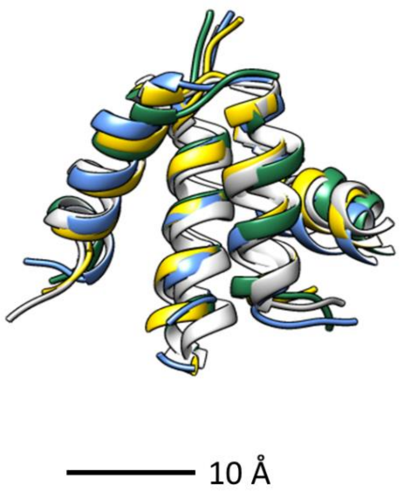

Supplementary Figure S4. Superimposed structures of p55 (yellow), Penicillium chrysogenum virus (blue), Saccharomyces cerevisae virus L-A (white), Omono river virus (green) capsid proteins.

p-AD	p-BD	Yeast Growth					
		DDO	QDO	QDOXA + 3-AT			
				0 mM	1 mM	2.5 mM	5 mM
T	53						
T	Lam						
	Empty						
DNAJ heat shock family protein (AT2G22360)	ORF1 961-1200						
	ORF1 320-670						
	Empty						
Ribosomal protein L17 family protein (AT3G54210)	ORF1 961-1200						
	ORF1 320-670						
	Empty						
Sec14p-like phosphatidylinositol transfer family protein (AT1G72160)	ORF1 961-1200						
	ORF1 320-670						
	Empty						
GDSL-like Lipase/Acylhydrolase superfamily protein (AT5G45670)	ORF1 961-1200						
	ORF1 320-670						
	Empty						
Chloroplast GRX 12, GRXS12 (AT2G20270)	ORF1 961-1200						
	ORF1 320-670						
	Empty						
Chaperone protein dnaJ-like protein (AT5G06130)	ORF1 961-1200						
	ORF1 320-670						
	Empty						
GPI-anchored protein (AT3G18050)	ORF1 961-1200						
	ORF1 320-670						
	Empty						
Pyrimidin 4 (PYR4) (AT4G22930)	ORF1 961-1200						
	ORF1 320-670						
	Empty						
Pectinacetylesterase family protein (AT4G19420)	ORF1 961-1200						
	ORF1 320-670						
	Empty						
Double Clp-N motif protein (AT4G12060)	ORF1 961-1200						
	ORF1 320-670						
	Empty						
PEBP (phosphatidylethanolamine-binding protein) family protein (FT) (AT1G6548)	ORF1 961-1200						
	ORF1 320-670						
	Empty						
Clone RAFL09-89-G08 (R19778) putative cellulose synthase catalytic subunit (RSW1) (At4g32410)	ORF1 961-1200						
	ORF1 320-670						
	Empty						
mRNA for plastid protein, complete cds, clone: RAFL15-06-D14 (AT1G32580.1)	ORF1 961-1200						
	ORF1 320-670						
	Empty						
Papain family cysteine protease (AT4G16190)	ORF1 961-1200						
	ORF1 320-670						

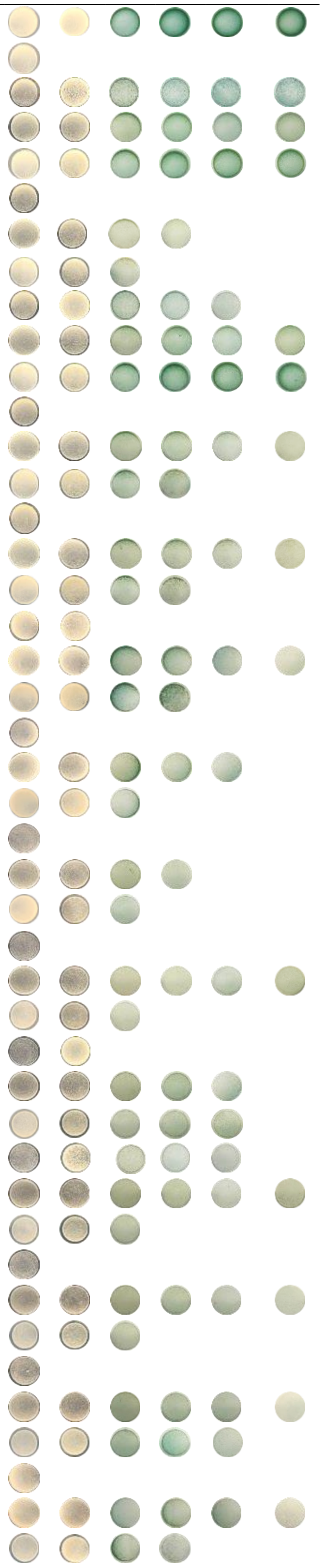

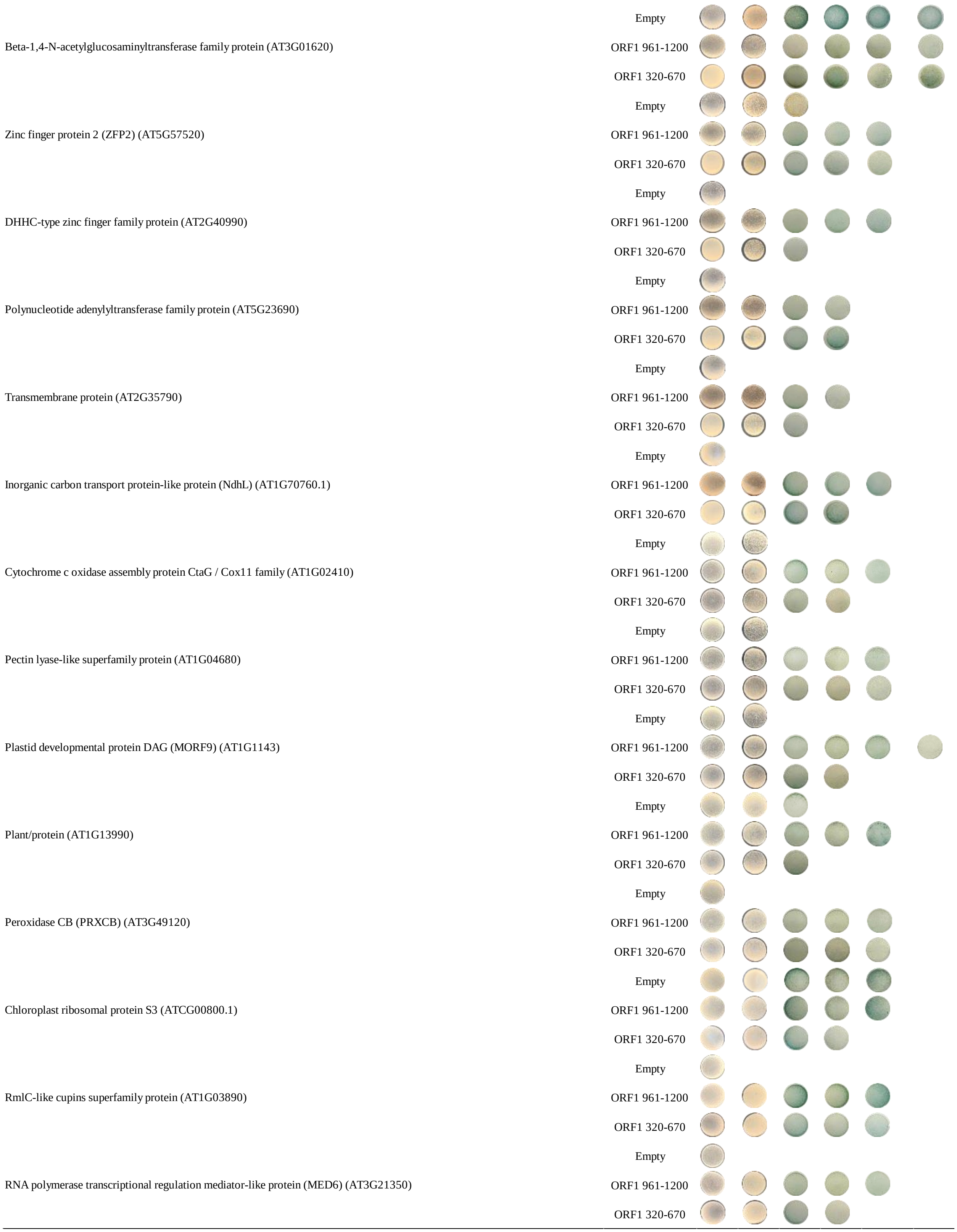

Supplementary Figure S5. Spot plating shows the validation of genuine positives interacting with CP2 and CP4.

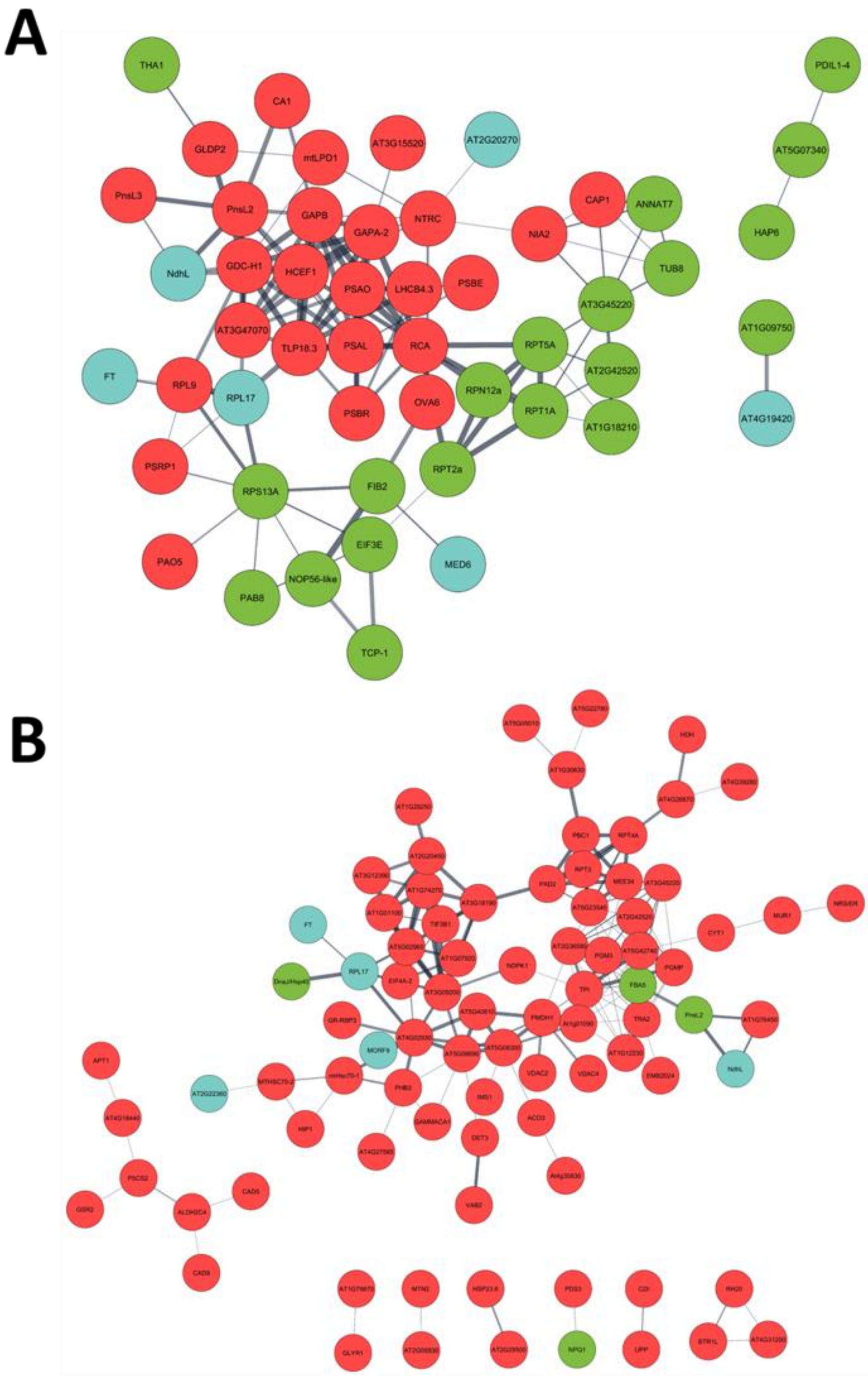

Supplementary Figure S6. Protein-protein interaction network (PPI) of *Carica papaya* differentially accumulated proteins during papaya meleira virus (PMeV) complex infection and PMeV CP2 and CP4-interacting proteins;
